# Novel Crosslinked Anion Exchange Membranes Based on Thermally Cured Epoxy Resin: Synthesis, Structure and Mechanical and Ion Transport Properties

**DOI:** 10.3390/membranes14060138

**Published:** 2024-06-11

**Authors:** Daniil Golubenko, Farah Ejaz Ahmed, Nidal Hilal

**Affiliations:** New York University Abu Dhabi Water Research Center, New York University Abu Dhabi, Abu Dhabi P.O. Box 129188, United Arab Emirates; dg4300@nyu.edu (D.G.); farah.ahmed@nyu.edu (F.E.A.)

**Keywords:** anion-exchange membranes, epoxy resin, thermal curing, diffusion dialysis, electrodialysis

## Abstract

Limitations in existing anion exchange membranes deter their use in the efficient treatment of industrial wastewater effluent. This work presents an approach to fabricating novel anion-conducting membranes using epoxy resin monomers like hydrophobic or hydrophilic diglycidyl ether and quaternized polyethyleneimine (PEI). Manipulating the diglycidyl ether nature, the quantitative composition of the copolymer and the conditions of quaternization allows control of the physicochemical properties of the membranes, including water uptake (20.0–330%), ion exchange capacity (1.5–3.7 mmol/g), ionic conductivity (0.2–17 mS/cm in the Cl form at 20 °C), potentiostatic transport numbers (75–97%), as well as mechanical properties. A relationship was established between copolymer structure and conductivity/selectivity trade-off. The higher the quaternized polyethyleneimine, diluent fraction, and hydrophilicity of diglycidyl ether, the higher the conductivity and the lower the permselectivity. Hydrophobic diglycidyl ether gives a much better conductivity/selectivity ratio since it provides a lower degree of hydration than hydrophilic diglycidyl ether. Different mesh and non-woven reinforcing materials were also examined. The developed membranes demonstrate good stability in both neutral and acidic environments, and their benchmark characteristics in laboratory electrodialysis cells and batch-mode dialysis experiments are similar to or superior to, commercial membranes such as Neosepta© AMX, FujiFilm© Type1, and Fumasep FAD-PET.

## 1. Introduction

Wastewater that is rich in residual acids and heavy metals is produced in many industries, including metallurgy, electroplating, paper milling and mining [1]. Rapid industrial growth in recent years translates into increasing wastewater production with large amounts of toxic waste and environmental pollution. When disposed of without treatment, this wastewater has severe effects on human health and local biodiversity [2]. Treatment is necessary for safe disposal and for the potential recovery of metal ions and acids from the acidic stream. Conventional processes used to treat industrial acidic effluent include neutralization [3], precipitation [4] and solvent extraction [5]. Membrane separation processes such as electrodialysis (ED) and diffusion dialysis (DD) offer several advantages over other methods, such as compact design, simple operation, low capital requirements, environmental friendliness and low energy consumption [6,7,8,9,10,11]. DD is a promising technology for the separation and concentration of various types of acids. DD relies on diffusion through an ion-exchange membrane in the presence of a concentration gradient. The conductance of anions in the matrix of anion-exchange membranes (AEMs) is high due to the high concentration of fixed cationic groups in the membrane matrix. In turn, the conductance of cations such as H^+^ or M^n+^ is reduced due to electrostatic repulsion from the positively charged membrane matrix-Donnan exclusion. As a result, diffusion of cations is preferential towards low valence charge and high mobility, which provides high selectivity to acid compared to heavy metal salts. An ideal AEM for DD should simultaneously possess a high proton dialysis coefficient and high H^+^/M^n+^ separation factor, as the former determines acid recovery efficiency while the latter is an indicator of the purity of the recovered acid product [12]. AEMs are also employed in electrodialysis (ED) modules, which facilitate the removal of ions from wastewater and/or brackish water streams. In an ED module, an applied potential drives the migration of ions selectively through ion exchange membranes (IEM). Suitable AEMs for these processes exhibit high ionic conductivity, high permselectivity, superior mechanical strength, high chemical stability, low swelling, and ease of fabrication [13]. However, anion exchange membranes often have such disadvantages as high manufacturing costs caused by the need for toxic chemicals, low stability in alkaline media and a less effective conductivity/selectivity ratio [14,15,16]. Therefore, the search for new and modification of anion-conducting materials has continued [17].

Epoxy resins are an abundant material used in construction due to their resilience and ease of use [18]. They are synthesized by thermally curing a diepoxide and a crosslinking agent such as polyamine through an epoxy-ring opening reaction, which creates a dense polymer matrix with remarkable mechanical and chemical resistance [19,20]. To the best of our knowledge, PEI-based epoxy resins have never been used to fabricate anion-conducting membranes. In this work, we use amine fragments in the epoxy resin as a polyelectrolyte to provide anion-conducting properties. Epoxy resins are practically not solvated in water due to the high content of hydrophobic bisphenol A diglycidyl ether (BADGE). In addition, the polyamine in the epoxide structure contains uncharged secondary or tertiary amino groups that cannot provide anion transport. To optimize the epoxy resin structure for ion transport we suggest the next steps: improve hydrophilicity by increasing the content of polyamine and using a more hydrophilic diepoxide such as poly(propylene glycol) diglycidyl ether (PPGDGE); introduce positively charged functional groups by PEI quaternization; improve swelling capability by adding inert diluents as pore-forming agent, which would be washed out after thermal curing.

Membrane formation can be performed by impregnating some reinforcing matrix, such as polymer mesh or non-woven material, with subsequent lamination and thermal curing. The reinforcing material is necessary to form a uniformly thin film from the initial mixture of liquid components, which will be left after curing. A classical Menshutkin reaction with methyl iodide can carry out the quaternization of PEI after curing. The advantages of such membranes are the simplicity of synthesis, availability of reagents and minimal use of organic solvents. The disadvantage of the proposed synthesis scheme is the use of methyl iodide, which requires high safety standards due to its toxicity, and the presence in the structure of bisphenol A, an industrial chemical produced in large quantities for use in manufacturing plastics. However, the adverse effects of bisphenol A on human health drive the development of safer and more sustainable alternatives such as Para, para’-bis guaiacol [21].

There are a few studies that use epoxy resins to fabricate membrane materials in reported literature. R. Verbeke and coauthors developed chlorine-resistant epoxide-based membranes for baromembrane water desalination by interfacial polymerization [22]. K. Sakakibara and coauthors proposed a method to prepare an epoxy resin-based monolithic membrane without a surface skin layer for lithium-ion battery application [23]. S.K. Jeong used glycidyl methacrylate as an additive to the copolymer of vinylbenzyl chloride and divinylbenzene, which improved the thermal stability of the anion-conducting membranes for fuel cells [24]. Much more often, epoxide groups are used for the modification of polymers and polymer composites as part of membranes due to their ability to enter into reactions involving the opening of the epoxide [25,26,27,28,29]. 

The aim of this work is to develop anion-conducting membranes based on epoxy resin precursors and to understand how the degree of hydration, ion exchange capacity, conductivity, permselectivity and mechanical properties depend on the copolymer composition, reinforcing material and synthesis conditions. The study will also evaluate the applicability of these membranes to diffusion dialysis for nitric acid recovery and electrodialysis.

## 2. Materials and Methods

### 2.1. Chemicals and Materials

Polyethylenimine (branched average Mw ~800 by LS, average Mn ~600 by GPC, Sigma-Aldrich CHEMIE GmbH, Steinheim, Germany), Bisphenol A diglycidyl ether (Sigma-Aldrich CHEMIE GmbH, Steinheim, Germany), Poly(propylene glycol) diglycidyl ether (average Mn 380, Sigma-Aldrich CHEMIE GmbH, Steinheim, Germany), 1-Methyl-2-pyrrolidinone (EMPLURA^®^, Sigma-Aldrich Sigma-Aldrich CHEMIE GmbH, Steinheim, Germany), Methyl iodide (stabilized with copper wire, for synthesis, SDFCL, Mumbai-30, India), Acrylonitrile (LiChrosolv^®^, Sigma-Aldrich CHEMIE GmbH, Steinheim, Germany), acetone (EMSURE^®^, Sigma-Aldrich CHEMIE GmbH, Steinheim, Germany), chloroform (EMSURE^®^, Sigma-Aldrich CHEMIE GmbH, Steinheim, Germany), sodium chloride (≥99.5%, Analytical reagent grade, Fisher Chemical, Geel, Belgium), sodium sulfate (ACS Reagent, ≥99.0%, anhydrous, Sigma Aldrich CHEMIE GmbH, Steinheim, Germany), Sodium Nitrate (ACS Reagent, ≥99.0%, anhydrous, Sigma Aldrich CHEMIE GmbH, Steinheim, Germany), silver nitrate (≥99.9%, Analytical reagent grade, Fisher Chemical, Geel, Belgium), phenolphthalein (General purpose grade, Fisher Chemical, Geel, Belgium), potassium chromate (Sigma Aldrich CHEMIE GmbH, Steinheim, Germany), potassium hydrogen phthalate (SDFCL, Mumbai-30, India), zinc nitrate hexahydrate (reagent grade, 98%, Sigma Aldrich CHEMIE GmbH, Steinheim, Germany), cobalt nitrate hexahydrate (purified, SDFCL, Mumbai-30, India), potassium carbonate (for analysis, EMSURE^®^, Sigma Aldrich CHEMIE GmbH, Steinheim, Germany), Type 1 and Type 2 water was obtained with Milli-Q IQ 7015 Milli-Q (Merck Millipore, Burlington, MA, USA).

Five different materials were tested for reinforcement: three types of meshes from polyamide (PA), polyethylene terephthalate (mPET) and polypropylene (mPP), two types of non-woven fabrics from polypropylene (nwPP) and polyethylene terephthalate (nwPET). All materials were ordered from local suppliers, and the conformity of the polymers to the declared ones was verified by FTIR spectroscopy. Several commercial anion exchange membranes were also tested for comparison of properties: RALEX© AM-PP (MEGA a.s., Stráž pod Ralskem, Czech Republic), Neosepta© AMX (ASTOM Co., Tokyo, Japan), FujiFilm© T1 (FUJIFILM, Minato, Japan) and Fumasep© FAD-PET-75 (FuMA-Tech, Bietigheim-Bissingen, Germany).

### 2.2. Membrane Fabrication

**Figure 1:**
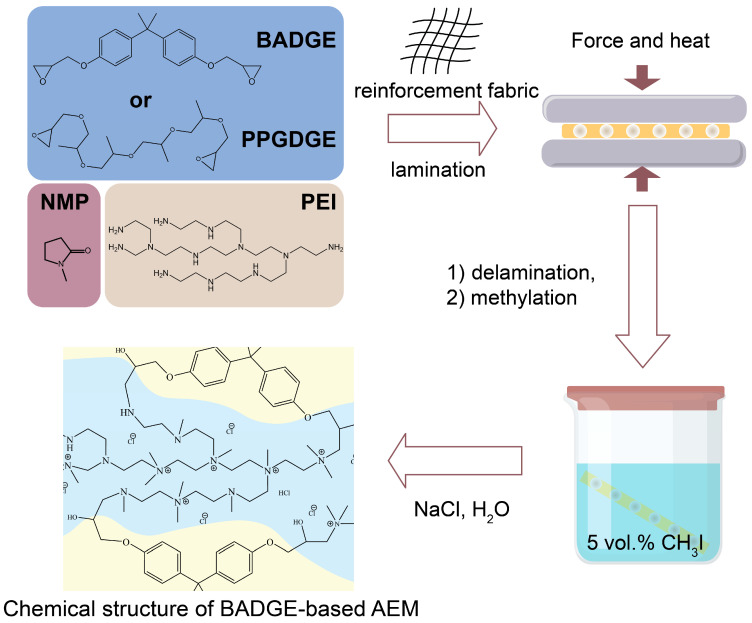
Membrane fabrication scheme and final chemical structure of BADGE-based membranes.

**Figure 2:**
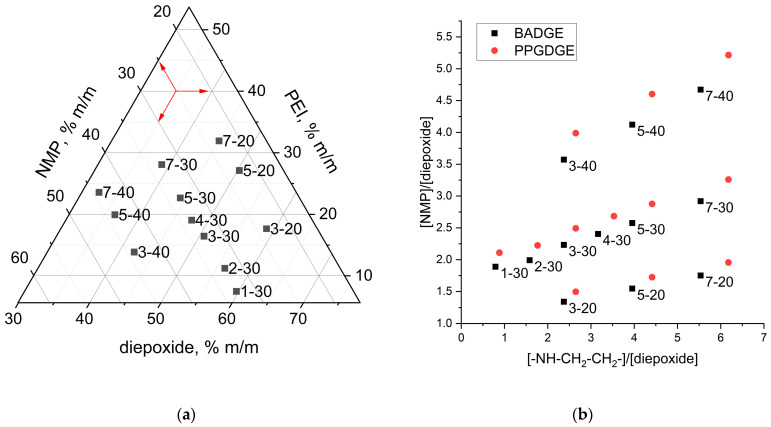
Mass (**a**) and molar (**b**) content ternary graph for compositions under study.

Membranes were prepared according to the scheme shown in Figure 1. About 2 mL of a mixture of Bisphenol A diglycidyl ether (BADGE) or Poly(propylene glycol) diglycidyl ether (PPGDGE), polyethyleneimine (PEI), 1-Methyl-2-pyrrolidinone (NMP) was prepared in an Eppendorf, all reagents were weighed on an analytical balance. The mass and molar composition of the ternary mixtures with labels under study are presented in Figure 2. After mechanical stirring, the mixture was kept in a 0.2 bar vacuum in a vacuum oven (VT 6060 P-BL, Thermo Scientific, Waltham, MA, USA) for 5–10 min to remove air bubbles. The reaction mixture was then poured into a zip-lock bag (11 cm × 8 cm), where a reinforcing mesh/fabric was placed beforehand. After removing the excess mixture and squeezing out visible air bubbles, the zip-lock bag was closed and clamped between two sheets of tempered glass (102 mm × 102 mm × 5 mm) using clamps. Thermal curing was carried out in an oven (DRY-Line 56 Prime, VWR Inc., West Chester, PA, USA) at a constant temperature of 80 °C for a certain time, which was determined upon termination of the reaction in a separate experiment using differential scanning calorimetry (DSC 214 Polyma, Netzsch, Selb, Germany). The influence of curing temperature on membrane properties was also investigated by varying curing temperature at 50, 60, 70 or 80 °C. After the curing step the PEI quaternization reaction to create positively charged functional groups was performed using 50 mL of a 5% *v/v*. solution of methyl iodide in one of the following solvents: acetone (ACE), acrylonitrile (AN), *N*-methylpyrolidone (NMP), and chloroform (TCM). Also, approximately 1 g of potassium carbonate was added to the alkylation mixture to neutralize the acid released during the Menshutkin reaction. 

The alkylation was carried out for 12 h in a sealed container under stirring on an orbital shaker (LAB FISH^®^), and all work with methyl iodide was carried out in a fumehood due to its toxicity. After alkylation, the membranes were placed for 5–10 h in a solution of 5–6 M NaCl with the addition of potassium carbonate (about 1% m/m) to hydrate the membranes and decompose methyl iodide residues. The membranes were washed in 1–2 M sodium chloride solution for a day with periodic solution changes (3–4 times) and then in Type 1 deionized water with periodic changes (3–4 times) for the next day. In the long term, the membranes were stored in 2–3 M NaCl and equilibrated with the required solutions before experiments.

Different ratios of reactants were investigated in this study to determine the influence of the composition of the initial ternary mixture on the membrane properties. Figure 2 shows the labeling and mass/molar composition of the ternary mixtures. Typical component masses can be found in Table S1. The labels of the ternary reaction mixtures consist of two symbols X-Y, where X is a number depending on the mass ratio of epoxide to amine (“1”—1:0.1, “2”—1:0.2, “3”—1:0.3, and so on), and Y is a number depending on the mass fraction of NMP diluent in the mixture (“20”—23%, “30”—33%, “40”—44%). The higher X is, the more amine is in the mixture. If it is not apparent from the context, the name of the diepoxide used (BA—Bisphenol A diglycidyl ether, PPG—Poly(propylene glycol) diglycidyl ether) was added before the designation of the composition, and after that, the designation of the reinforcing matrix used (mPP—polypropylene mesh, nwPP—non-woven polypropylene, and so on), or the type of solvent in which the membrane was alkylated (AN—acrylonitrile, NMP—*N*-methylpyrollidone, ACE—acetone, TCM—trichloromethane) was added. For example, the designation BA_30-2_PA_AN denotes a membrane derived from bisphenol A diglycidyl ether, with the composition of reactants 30-2, polyamide mesh was used as reinforcing material, and alkylation was carried out in a solution of methyl iodide in acetonitrile. However, we tried to use such complete designations due to their complexity rarely.

### 2.3. Membrane Structure and Composition

The structure and composition of the fabricated membranes were characterized using various methods. FTIR spectra were obtained using Nicolet™ iS™ 5 (Thermo Scientific, Waltham, MA, USA) with ID7 ATR (64 scans, 2 cm^−1^ resolution). Membrane morphology was studied using a Scanning Electron Microscope (SEM)–ThermoFischer Quanta3D (Thermo Scientific, Waltham, MA, USA) with energy dispersive X-ray spectroscopy (EDS) detector (Oxford Instruments, High Wycombe, UK). Membrane cross-sections were prepared by cutting the samples cooled down to liquid nitrogen temperature. Before imaging, the samples were sputter-coated with a 30 nm gold film to reduce charging effects with the help of 108 Auto Sputter Coater (Ted Pella, Redding, CA, USA). Images were obtained in backscattered electron mode at a 5 keV accelerating voltage, and EDS-SEM studies were conducted at a 10 keV accelerating voltage.

### 2.4. Membrane Water Uptake and Ion Exchange Capacity

Water uptake and ion exchange capacity constitute key properties of ion exchange membranes; these parameters are determined by the physicochemical structure of the membrane and largely determine its transport characteristics. To measure water uptake (WU), the sample of membrane equilibrated with Type II was weighed using an analytical balance (Cubis^®^ MCA3203S-2S00-E, Sartorius AG, Gottingen, Germany) before and after dehydratation at 70 °C during 4–6 h in the laboratory oven (AX Oven, CARBOLITE GERO, Sheffield, UK). The value of WU was calculated using the following equation:

(1) $$WU=\frac{m_{w}-m_{d}}{m_{d}}\cdot 100\%$$

where m_w_ and m_d_ are the hydrated and dry membrane masses, respectively.

To measure the strong base ion exchange capacity (s-IEC), the dry preweighed membrane sample was equilibrated with an excess of 1 M of sodium nitrate solution. Then, the concentration of the exchanged chloride ions was determined by argentometric titration with potassium chromate as an indicator. The s-IEC was calculated using the following equation:

(2) $$s{IEC}_{\;}=\frac{V(NaNO_{3})\cdot C({Cl}^{-})}{m_{d}},\;\mathrm{mmole}\;g^{-1}$$

where V(NaNO_3_) is the volume of sodium nitrate solution, C(Cl^−^) is the concentration of chloride ions determined by argentometric titration, and m_d_ is the mass of the dry membrane.

The acid–base back titration method was used to measure weak base ion exchange capacity (w-IEC). This method is based on the neutralization of weakly basic amino groups in the membrane with hydrochloric acid. To do this, a weighed dry sample of the membrane was placed in approximately two-fold excess of 0.01 M hydrochloric acid and equilibrated for 12–14 h with stirring. Then, the concentration of unreacted acid was determined by titration with a 0.01 M alkali solution with a phenolphthalein indicator. The alkali solution was standardized by titration of a standard solution of potassium hydrogen phthalate. The w-IEC was calculated using the following equation:

(3) $${wIEC}_{\;}=\frac{V(HCl)\cdot ({C\left(HCl\right)}_{0}-{C\left(HCl\right)}_{1})}{m_{d}},\;\mathrm{mmole}\;g^{-1}$$

where V(HCl) is the volume of 0.01 M hydrochloric acid added to the membrane sample, and C(HCl)_0_ and C(HCl)_1_ are the concentration of acid before and after equilibration with the membrane.

All used capacity and water uptake values were corrected for the reinforcement content. This is important because the content of the reinforcing mesh is not constant for different materials. To calculate the polymer content (PC), a rectangular sample of membrane and mesh with dry area S was weighed, and the copolymer content (PC) was calculated using the following formula:

(4) $$PC=1-\frac{m_{r}/S_{r}}{m_{m}/S_{m}},\;\%$$

where m_r_ and m_m_ are masses of reinforcing material and membrane, S_r_ and S_m_ are areas of reinforcing material and membrane. The capacity and water uptake were corrected by dividing by the PC copolymer fraction. The polymer fraction for all membranes was in the range of 0.70–0.80.

### 2.5. Differential Scanning Calorimetry

The curing process was analyzed by DSC using the Netzsch DSC 214 Polyma instrument (Netzsch, Selb, Germany) to determine the time required for the reaction end and to estimate the fraction of epoxy-ring opening. The study was conducted in isothermal mode at the same temperature as the curing temperature, with a heating rate of 80 K/min and a sample mass of 10–15 mg. The DSC curve obtained under such conditions during the first few minutes of measurement contains significant thermal fluctuations associated with rapid heating and attaining steady-state conditions. To deduct these thermal fluctuations, the measurement under similar conditions was repeated on the same sample after curing, and the obtained curve was subtracted from the DSC curve of the non-cured sample.

### 2.6. Mechanical Properties

Stress—strain experiments were performed using a Universal Testing System 5960 series (Instron, Norwood, MA, USA) with a force sensor at 500 N under room conditions (RH = 40–60% and 20–22 °C). Rectangular membrane samples 60 mm in length and 10 mm in width were pre-equilibrated in Type I water (three to six samples were tested for each membrane). The thickness (T) of each sample was taken as the average values of three points measured before the experiment by a micrometer. The gauge length (GL) of the samples was adjusted to 30 mm; the test rate was set to 3 mm/min with a pre-load of 0.5 N. The variation of force (F) with displacement (d) was recorded and exported from the instrument. Stress and strain were then determined using the following equations:

(5) $$Stress=\frac{F}{T*w},\;\mathrm{MPa}$$

(6) $$Strain=100\cdot \frac{d}{GL},\%$$

Deformation at break (%), strength at break (MPa), Young’s modulus (MPa), and yield strength (MPa) were calculated and averaged from stress–strain curves for every sample. Young’s modulus was calculated as the slope of linear dependence in reversible deformations range 0–3%. Yield strength was found as the intersection of the linear regions corresponding to the reversible and irreversible deformations.

### 2.7. Membrane Ionic Conductivity, Permselectivity and Salt Diffusion Permeability

Key transport characteristics of membranes are ionic conductivity and permselectivity. The ionic conductivity of the membranes (σ, mS/cm) in chloride form was measured by sandwiching each membrane (active area: 0.785 cm^2^) between two soft graphite foil and two stainless steel electrodes by impedance spectroscopy using a Autolab PGSTAT302N potentiostat/galvanostat (Metrohm, Herisau, Switzerland) over a frequency range of 1 MHz to 100 Hz. The system was immersed in type I water at room temperature (20–22 °C). To exclude the cell contribution, the impedance of the short-circuited cell was measured and subtracted from the impedance of the cell with the membrane following the approach described previously [30]. Ionic resistance was defined as the intercept of the active impedance axis on the Nyquist plots. The following equation was used to calculate the membrane conductance:

(7) $$\sigma =\frac{T}{RS},\;\mathrm{mS}\;{\mathrm{cm}}^{-1}$$

where T is the membrane thickness, R is the membrane ionic resistance, and S is the active area of the cell.

**Figure 3:**
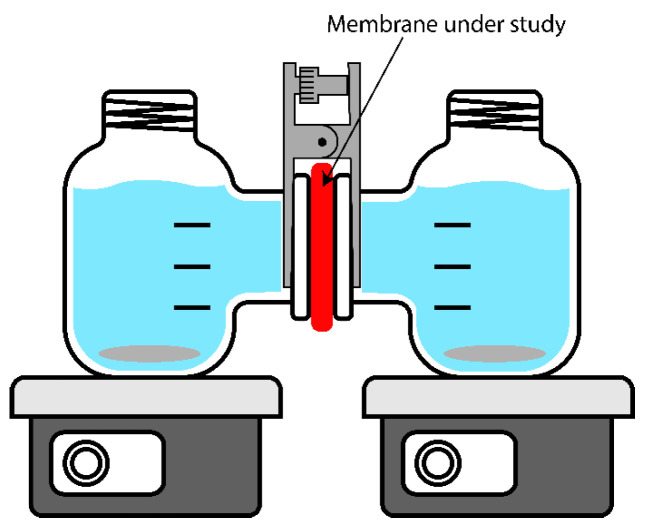
Schematic of an H-cell to characterize membrane potential and diffusive permeability (electrodes and sensors not depicted).

Two parameters were used to characterize permselectivity. First, the potentiometric transport numbers (t_pot_, %) were calculated from membrane potential (E_m_)–potential difference measured with the help of two Ag/AgCl reference electrodes between two compartments of H-cell (MFC Reactor Device for 100 mL Microbial Fuel Cell) filled with 0.5 and 0.1 M NaCl solutions and separated by a membrane under study. The setup scheme is shown in Figure 3. The solutions were actively stirred using magnetic stirrers; the experiment was carried out at room temperature. Correction on junction potential difference was made according to [31]. Detailed calculation and methodology of determination of potentiometric transport are described in [32].

The second parameter characterizing membrane permselectivity was sodium chloride diffusion permeability, which was measured by the rate of sodium chloride diffusion from the chamber with higher concentration into the chamber with pure water in the H-cell described above. Type II water and a conductometric sensor were placed in one chamber at the beginning of the experiment, and 0.1 M sodium chloride solution was placed in the second chamber. To find the sodium chloride flux, the time dependence of the conductivity of the solution was determined using a Jenway conductometer (3540, Cole-Parmer Ltd., Vernon Hills, IL, USA). The conductivity values were converted to concentration values using the calibration. The diffusion permeability coefficient was calculated using the following formula:

(8) $$P_{NaCl}=\frac{dC}{dt}\cdot \frac{V}{S}\cdot \frac{T}{C^{0}},\;{\mathrm{cm}}^{2}\;s^{-1}$$

where dC/dt is the rate of increase of sodium chloride concentration in the chamber with pure water, V is the volume of solution in this chamber (140 mL), S is the active area of the membrane (6.97 cm^2^), and C^0^ is the sodium chloride concentration in the chamber with sodium chloride solution, the change of which did not exceed 1% during the experiment, therefore it was taken as a constant value of 0.1 M.

### 2.8. Membrane Voltammetric Characterization

**Figure 4:**
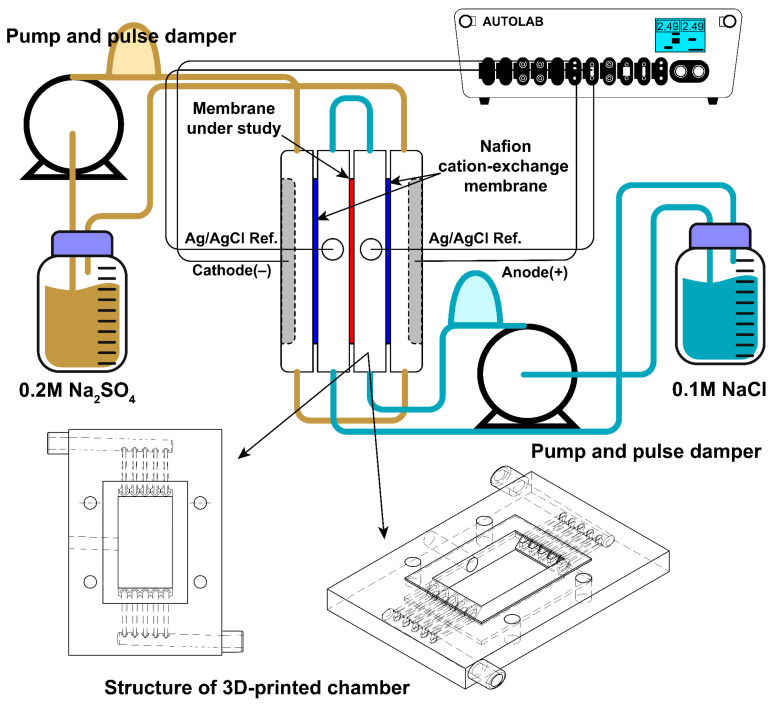
Scheme of the setup and cell compartment for the current-voltage characteristic measurement.

To characterize membranes during the desalting process of sodium chloride solution, the current-voltage characteristics (CVC) of a laboratory electrodialysis cell built based on [33,34] were studied. The setup scheme is represented in Figure 4. The compartments are divided by membranes, consisting of a 3D-printed plastic compartment, and two cut silicone gaskets with an effective membrane area of 1.75 cm × 3.00 cm. 3D-printed plastic compartments were designed to provide uniform flow distribution and printed using a 3D printer (J750, Stratasys, Eden Prairie, MN, USA). 3D models used to print the cell compartment have been added to supplementary data. The thickness of the compartment is 6 mm. The total thicknesses of silicone gaskets are 0.6 mm. Hence, the distance between the membranes (h) is about 6.6 mm. The compartments adjoining the membrane under study are separated from the electrode compartments by a Nafion© (Chemours company, Wilmington, DE, USA) cation-exchange membrane. Two platinum mesh electrodes were fixed in electrode compartments and used as cathode and anode for current polarization. Two silver/silver chloride electrodes (R0302, YPLZYANJIAO, China) with a diameter of 3.8 mm were inserted into compartments adjoining the membrane under study and were used to measure potential drop on the membrane. All compartments were pumped by peristaltic pumps (BT600FC, China), electrode and membrane compartments with 0.2 M sodium sulfate and 0.1 M sodium chloride with a flow rate of 300 mL/min and 50 mL/min, respectively. To provide laminar uniform solution flow, the Masterflex^®^ Pulse Dampeners for L/S^®^ and I/P^®^ Tubing, Avantor^®^ (VWR Inc., West Chester, PA, USA) were connected between the outlet of the pump and the inlet of the cell for both sodium chloride and sodium sulfate solutions. The latter is important to simplify the mathematical description of ion transport in the cell. A Potentiostat/Galvanostat Metrohm Autolab PGSTAT302N was used to measure CVC with a constant scan rate 5 × 10^−4^ A/s in the 0–4.0 V range.

To estimate the theoretical limiting current density, the following formula was used [34]:

(9) $$i_{lim}=1.47\cdot \frac{FDc^{0}}{h(T_{1}-t_{1})}\cdot {\left(\frac{h^{2}V}{LD}\right)}^{1/3},\;\mathrm{mA}\;{\mathrm{cm}}^{-2}$$

where D is the salt diffusion coefficient (1.65 × 10^−5^ cm^2^/s), c^0^ the inlet concentration (0.1 M), h the distance between the membranes (0.66 cm), V the average linear solution velocity (0.72 cm/s), L the length of the membrane active area (3 cm), T_1_ and t_1_ the salt counter-ion effective transport number in the membrane (T_1_ = 1.0) and the transport number in the solution (t_1_ = 0.604), respectively, and F the Faraday constant (96,485 C/mole).

### 2.9. Diffusion Dialysis Acid Recovery

The diffusion dialysis experiment was also performed in the H-cell (Figure 3). A solution containing 5% m/m HNO_3_, 500 ppm Co (Co(NO_3_)_2_) and 500 ppm Zn (Zn(NO_3_)_2_) was poured into one of the chambers, and water was poured into the second chamber. In the chamber with pure water, conductivity was measured periodically using a conductometer. Conductivity values were converted to nitric acid concentration using the calibration dependence of the conductivity of nitric acid solutions on concentration. The contribution to the conductivity of cobalt and zinc salts was neglected because, firstly, their flux is at least ten times less than that of nitric acid. Secondly, the specific conductivity of nitric acid is an order of magnitude higher than the specific conductivity of Co(NO_3_)_2_ or Zn(NO_3_)_2_. The experiment was carried out for at least 30 min in the case of highly permeable membranes or until the conductivity increased to 2–3 mS/cm in the case of low permeability membranes. Typical time dependences of acid concentration are given in Figure S1. Permeate was analyzed for cobalt and zinc content by ICP-MS spectrometry (Agilent 7800 ICP-MS connected with SPS 4 autosampler, Agilent Technologies, Santa Clara, CA, USA). Since the degree of dialysis was less than 5% of the initial value for all membranes, its change in the calculation of the permeability coefficient can be neglected, as well as the change in solution volumes. The acid permeability was calculated using a formula very similar to that for calculating the permeability of sodium chloride (7):

(10) $$P_{H}=\frac{dC_{H}}{dt}\cdot \frac{V_{p}}{S}\cdot {\left(\frac{C^{0}}{T}\right)}^{-1},\;{\mathrm{cm}}^{2}\;s^{-1}$$

where dC_H_/dt is the rate of acid concentration growth in the chamber with pure water, V is the volume of water in this chamber (140 mL), S is the active area of the membrane (6.97 cm^2^), C^0^ is the concentration of nitric acid in the feed solution, (0.794 M).

Metal permeability coefficients were estimated by the following formula, which differs from that in Equation (9) in that the rate of increase of metal concentration in this equation is calculated as the final concentration (C_M_) divided by the time of experiment (t):

(11) $$P_{Me}=\frac{C_{M}}{t}\cdot \frac{V_{p}}{S}\cdot {\left(\frac{C^{0}}{T}\right)}^{-1},\;{\mathrm{cm}}^{2}\;s^{-1}$$

S_H/Me_ selectivity coefficients were calculated as the ratio of P_H_/P_Me_ permeability coefficients.

## 3. Results and Discussion

### 3.1. DSC Study of Curing Reaction

**Figure 5:**
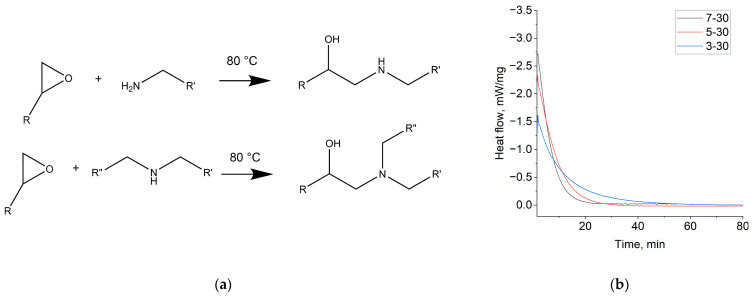
Reactions scheme between BADGE or PPGDGE epoxy groups and PEI amino groups (**a**); a typical isothermal curing DSC curves for some compositions of BADGE-PEI copolymers (**b**).

The polycondensation reaction of BADGE or PPGDGE and PEI is based on the epoxy-ring opening reaction by primary and secondary amino groups of the PEI molecule (Figure 5a) [20]. As DSC data provide the heat release flow (Figure 5b), which is proportional to the number of reacting groups per unit mass of the reaction mixture, it is possible to determine the termination of the curing process and the integrated heat of the reaction (Table 1). For the PPGDGE-PEI copolymer, the DSC curves are similar and are presented in Supplementary (Figure S2). The curing temperature was also optimized in the range of 50–80 °C; at lower temperatures, the reaction was slower, but the choice of temperature did not significantly affect the membrane properties. Therefore, all further syntheses were carried out at 80 °C. Data on temperature optimization are given in Supplementary Materials (part 2.2, Table S2, Figure S3).

Several typical patterns for curing end time and the maximum heat release rate can be observed. First, the lowest reaction rate is observed for X-Y membranes with lower X value, i.e., lower PEI content, which is observed for both BADGE-PEI and PPGDGE-PEI copolymers. Second, the reaction slows down with increasing Y, which is the diluent fraction.

The reduction of reaction rate due to increasing diluent fraction is due to the reduction of the concentration of reacting molecules. However, the drop in the reaction rate with decreasing PEI concentration is not so obvious since there is a simultaneous increase in the epoxide concentration. The heterogeneity of PEI distribution in the solution can explain the higher sensitivity of reaction kinetic to PEI concentration. According to the PEI molecular weight M_n_ = 600 g/mol as stated by the manufacturer, there are approximately 14 -CH_2_-CH_2_-NH- monomeric fragments in one molecule, and considering that it is a branched polymer, the amino groups are in a compact globule. Due to steric limitations, the reactivity of amino groups outside and inside the globule is different, and the effective concentration (activity) of amino groups is lower than formally calculated from the amounts of substances. This agrees with another literature study [35], which shows that the reaction rate is lower with hyperbranched PEI than with aliphatic triamine.

Following the literature data, the enthalpy of the BADGE reaction with primary or secondary amines is close to 110 kJ/mol and varies slightly depending on the nature of the amine [36]. For most compositions studied, the heat of the process given per 1 mol of epoxide groups is 87–126 kJ/mol (Table 1), which suggests a high degree of epoxide opening. However, for copolymer PPG 1–30 with the lowest PEI content, the heats fall to 51, much lower than the average. This indicates a reduced degree of epoxide opening and degree of curing.

### 3.2. Membrane Morphology and Composition

#### 3.2.1. FTIR

**Figure 6:**
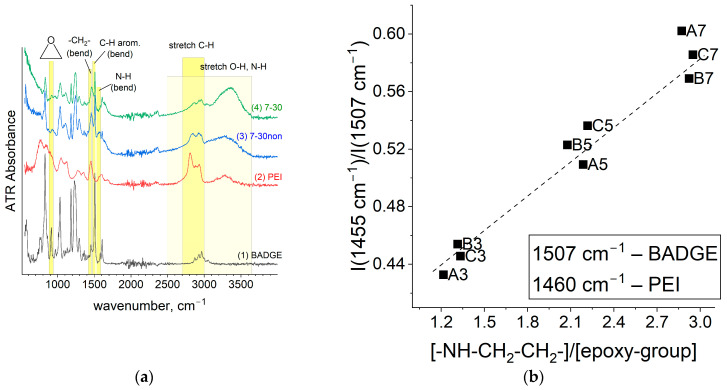

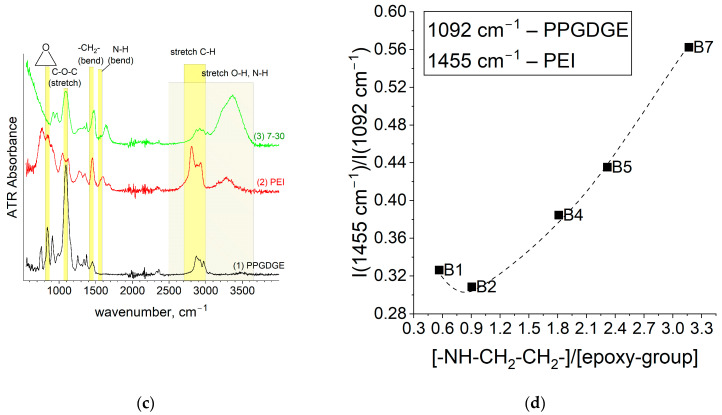
FTIR spectra of (**a**) BADGE-based and (**c**) PPGDGE-based membranes with initial PEI, BADGE and PPGDGE; correlations between the ratios of (**b**) 1460 and 1507 cm^−1^ or (**d**) 1455 and 1092 cm^−1^ and copolymer composition.

The FTIR spectra of membranes based on the copolymer BADGE-PEI (Figure 6a) and PPGDGE-PEI (Figure 6c) contain almost all vibrations characteristic of individual substances BADGE, PPGDGE and PEI. A key indicator of the opening of the epoxide ring is a sharp decrease in the intensity of signals 908 cm^−1^ for BADGE and 834 cm^−1^ for PPGDGE copolymers referred to characteristic valence vibrations of the C-O epoxide group [20,37,38].

Along with the change of the [-NH-CH_2_-CH_2_-]/[epoxy-group] ratio in the reaction mixtures, the 1455 and 1507 cm^−1^ vibrations characteristic of PEI and BADGE (Figure 6b), and 1455 and 1092 cm^−1^ vibrations (Figure 6d) characteristic of PEI and PPGDGE, respectively, change monotonously. Note that the PPG_1-30 membrane, for which the lowest degree of epoxide opening was observed, falls out of the described correlation. The observed deviation can be explained by the leaching out of part of the diepoxide with unreacted epoxy groups.

To track the changes in the spectrum after PEI quaternization, we also measured the FTIR spectrum of the unmethylated BA-7-30 membrane (Figure 6-“7-30non”). These changes were minor because the quaternary amino groups formed absorb weakly in the IR range, and their characteristic vibrations coincide with those of the tertiary, secondary, and primary amino groups. The proof of the alkylation reaction of amino groups is a significant decrease in the absorption intensity in the region 1544–1598 cm^−1,^ where the deformation vibrations of N-H bonds of secondary and primary amino groups are located. In addition, after alkylation, the structure of the broadened signal in the region 2900–3600 cm^−1^, where the valence vibrations of N-H and O-H are located, changes.

#### 3.2.2. Electron and Optical Microscopy, EDX Elemental Analysis

**Figure 7:**
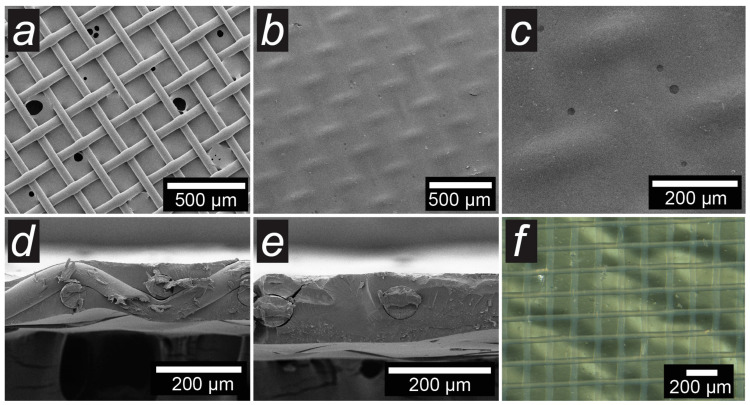
SEM image of the original PA mesh (**a**), surface (**b**,**c**) and cross-section (**d**,**e**) of the BA_5-30_PA membrane, and optical image (**f**) of the BA_5-30_PA membrane.

Figure 7 shows SEM images of the original polyamide mesh (a), the surface (b,c) and the cross-section (d,e) of the BA_5-30_PA membrane, as well as its optical image (f). The copolymer material completely covers the reinforcement mesh (Figure 7). SEM and optical images of the BA_5-30 membranes based on different reinforcement materials are shown in Figure S4. In all cases, the epoxy resin fills the available space of the reinforcement materials, and the surface texture of the membranes follows the texture of the original mesh/fabric. The copolymer material itself is a homogeneous transparent material, so the reinforcing mesh inside the membrane can be seen in optical images (Figure 7f).

A feature common to all membranes is worth noting–a bumpy texture with a characteristic width of 100–200 µm hollows. This texture is observed over the entire area of the sample. Most likely, the nature of this phenomenon is related to the processes of heat and mass transfer during curing but cannot be explained within the framework of this work. Additional SEM images illustrating the bumpy texture of the membranes are presented in the supplementary material (Figure S5).

SEM images of membranes based on PPGDGE-PEI copolymer look the same as those based on BADGE-PEI with similar reinforcing material (nwPP) and are presented in supplementary (Figure S6). The only thing worth noting is that the PPG_1-30 membrane is characterized by a high content of reinforcing material on the surface, which confirms the proposed above leaching of part of the copolymer material due to the low degree of opening of epoxy cycles.

**Figure 8:**
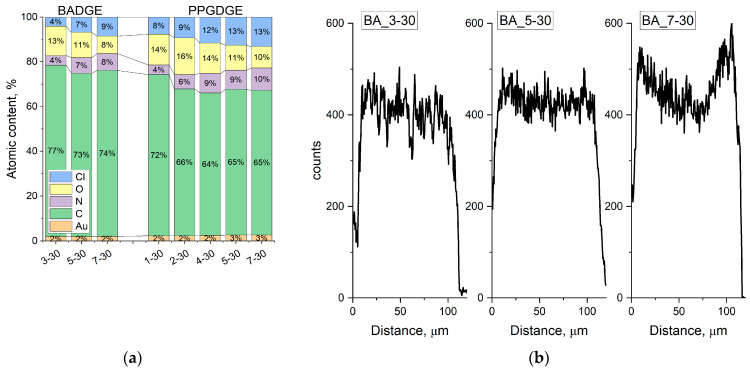
(**a**) SEM-EDS elemental composition of some membranes and (**b**) chlorine distribution across the membrane cross-sections.

SEM-EDS data (Figure 8a) shows that the membranes obtained based on both copolymers contain C, N, O, Au, and Cl. Au is observed because it was sputtered before SEM imaging to give the samples electronic conductivity. The presence of chlorine confirms the successful reaction of amino group quaternization—as chloride anion is the counter ion of R-N(Me)_3_^+^ Cl^−^ functional groups and appears in the membrane system when the membranes are converted to chloride form by sodium chloride solution. As the proportion of PEI increases, there is an increase in the chlorine and nitrogen content of quaternized PEI and a decrease in the oxygen content of BADGE or PPGDGE, consistent with FTIR spectroscopy data. Somewhat outside of the observed patterns is the behavior of the PPG-1-30 membrane, for which the carbon content is unusually high, and the oxygen content is low, which can be explained by the high proportion of reinforcing material on the surface observed in the SEM image analysis. The chlorine distribution along the cross-section of some BADGE-based membranes was also investigated (Figure 8b), as it can be seen that the methylation reaction leads to the formation of quaternary amino groups throughout the thickness of the polymer.

### 3.3. Ion-Exchange Capacity and Water Uptake

#### 3.3.1. Influence of Reaction Mixture Composition

**Figure 9:**
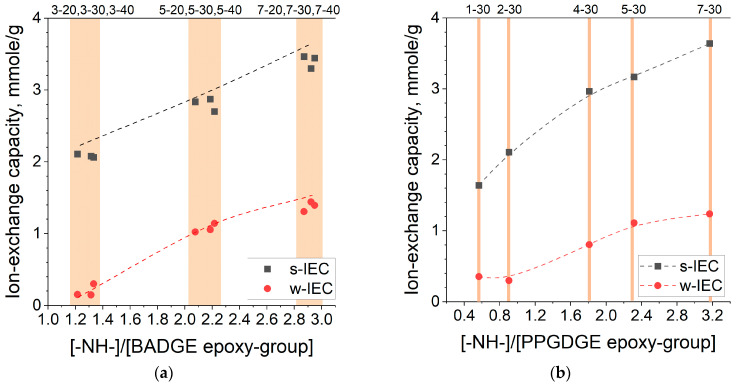
Correlation of capacity and [-NH-CH_2_-CH_2_-]/[epoxy-group] ratio in the reaction mixture for membranes based on BADGE (**a**) and PPGDGE (**b**).

The maximum theoretical strong-base ion-exchange capacity (s-IEC), which can be calculated from the amount of PEI added assuming 100% methylation, is between 2 and 6 mmol/g, depending on the composition of the reaction mixture. However, in practice, the s-IEC of both BADGE-based and PPGDGE-based membranes is in the range of 1.5–3.7 mmol/g (Figure 9). The reason is that quaternization yield is below 100%, and some of the amino groups remain as tertiary NR_3_ or secondary HNR_2_. Acid–base titration was conducted to confirm the weak base capacity presence (w-IEC). The higher the polyamine to diepoxide ratio in the reaction mixture, the higher s-IEC and w-IEC values and the greater the proportion of non-quaternized amino groups, which increases from 7–12 to 25–27% for BADGE and PPGDGE-based membranes. The diluent content has no effect on the ion exchange capacity.

While capacity dependence on polyamine content is apparent, the change in the proportion of non-quaternized amino groups is not. Since the reaction is heterogeneous, i.e., the alkylating agents diffuse in the swollen polymer, the first thing we paid attention to was the degree of swelling in the organic solvents (Table 2). As can be seen, after quaternization, the degree of swelling decreases by times for ACE, AN or by an order of magnitude for NMP, TCM, and in the case of water, it increases twofold. The likely reason for this is that quaternized amino groups, which are a close ionic pair R_2_N(Me)_2_^+^X^−^, solvate much worse by organic solvents than non-quaternized amino groups. Thus, as the alkylation reaction proceeds, the degree of swelling of the polymer decreases, the diffusion of the reactants slows down, and the reaction stops. By transferring this logic to the observed dependence of the degree of quaternization on the composition, we can expect that at a high content of uncharged diepoxides in copolymers, one can obtain higher degrees of alkylation than for membranes with a high PEI content because of the more significant diffusion limitation for the latter. The features of heterogeneous alkylation as a method of functionalization of ion-exchange polymers are hardly investigated in contrast to homogeneous alkylation.

**Figure 10:**
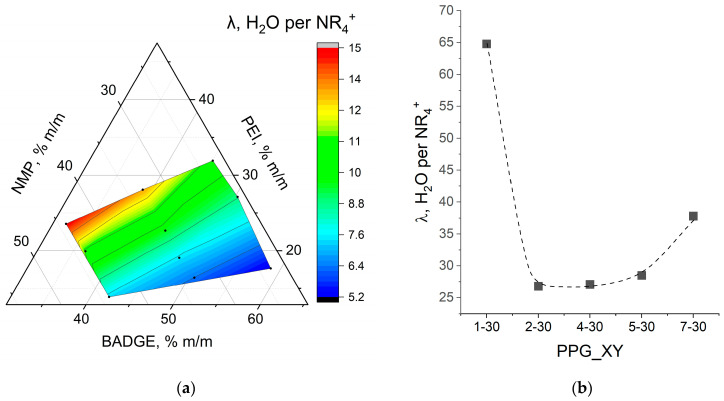
Relationships between hydration numbers and the composition of the reaction mixture based on BADGE (**a**) and PPGDGE (**b**).

Typically, for ion exchange membranes, the water content is compared regarding hydration numbers (λ), which is the number of water molecules per functional group, since water is mainly localized near hydrophilic functional groups. For BADGE-based membranes, λ is in the range of 5.2–15 and increases with the PEI and the diluent content (Figure 10). The dependence on PEI content can be attributed to shifting the copolymer composition towards hydrophilic PEI and simultaneously decreasing the content of hydrophobic BADGE. The second pattern is explained by the role of the diluent as a component of the reaction mixture responsible for forming free space. The addition of diluent can also be considered as a decrease in crosslinking density–the higher the diluent content, the more the system can expand during hydration. The described patterns are often observed in the correlation between the composition and properties of anion exchange membranes based on different polymers [39,40,41,42].

Hydration numbers for PPGDGE-based membranes are significantly higher than for BADGE based due to the feature of the chemical structure of PPGDGE, which consists of polypropylene glycol oligomer and thus is by nature much more hydrophilic and mobile than BADGE-containing hydrophobic aromatic groups. Leaving aside the dropout data for 1–30 membrane, for which the high degree of hydration is probably related to the low degree of polymerization, the water uptake and hydration numbers are higher the higher the PEI content. This correlation can also be explained by an increased proportion of the more hydrophilic quaternized PEI component.

#### 3.3.2. Influence of Synthesis Conditions

The solvent selection for alkylation was an important parameter affecting the fractions of weakly and strongly basic amino groups. When trying to obtain a series of BA_7-40_PA membranes based on rigid polyamide mesh alkylated in different solvents ACE, AN, TCM, and NMP, the membrane alkylated in NMP collapsed due to the detachment of the copolymer from the reinforcing material due to internal stresses, probably associated with a high degree of swelling. Membranes alkylated in NMP could be obtained only based on low-modulus reinforcing material like nwPP, which did not limit the geometrical changes of the copolymer due to swelling. In general, the s-IEC of membranes increases in the following series AN ≈ TCM >>> ACE ≈ NMP, with a maximum difference in the capacity of 1.1 mmol/g for 7–30 and 7–40 compositions (Table 3). This series correlates with the degree of swelling of the BADGE-PEI copolymer (Table 2), indicating the key role of swelling on the degree of quaternization. The exception is the membranes alkylated in TCM, which are characterized by relatively high swelling degrees but not by a high degree of quaternization. This may be explained by the fact that the depth of quaternization can be determined by the swelling of the epoxy copolymer and other unidentified factors.

### 3.4. The Ionic Conductivity and Permselectivity

#### 3.4.1. Influence of Reaction Mixture Composition

**Figure 11:**
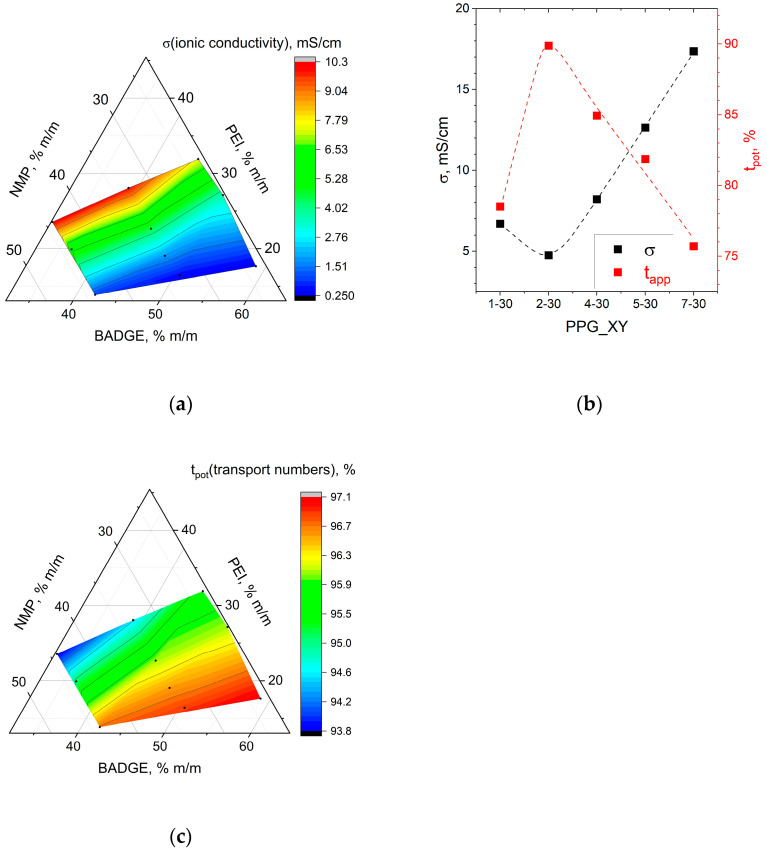
Correlation between Cl^−^-conductivity (**a**) and potentiostatic transport numbers (**b**) and the composition of the reaction mixture on the example of BA_XY_NYL (**a**,**b**) and PPG_XY_nwPP (**c**) membranes.

The higher the proportion of PEI and diluent, the higher the ionic conductivity (Figure 11). In general, the ionic conductivity of a system is proportional to the concentration of charge carriers and their mobility [43,44]. For the membranes under study, the number of charge carriers is determined by q-IEC capacity, which is affected by PEI content. The mobility of ions in the membrane matrix is also determined by the charge concentration, pore sizes, and the volume of the pore space filled with functional groups and water molecules, which depends on the water uptake. So, the observed correlations of conductivity and composition of the reaction mixture (Figure 11a,b) fit well into the described patterns for hydration number (Figure 10a,b) and capacity (Figure 9a,b).

The Donnan effect determines the permselectivity of ion exchange membranes, i.e., the exclusion of co-ions from the membrane matrix and the effective transport of counter-ions [43]. To determine the permselectivity of membranes, we used potentiostatic transport numbers (Figure 11b,c). The higher the proportion of PEI and diluent, the lower the permselectivity, which can be explained by increasing hydration number λ (the decrease in charge density) (Figure 10a,b). Also, the obtained data can be interpreted in terms of pore size [45,46]. The membrane permselectivity is determined by the electrostatic repulsion of co-ions from the pore walls. With increasing pore size, electrostatic repulsion decreases, allowing higher flux of non-selective co-ions.

#### 3.4.2. Influence of Synthesis Conditions

Although the choice of solvent at the quaternization step significantly affects ion exchange capacity, the effect on anion conductivity and transport numbers is weak (Table 3). The comparison of NMP and AN was performed for BA_7-30_mwPP membranes. Alkylation in AN provides a lower degree of quaternization and higher permselectivity than in NMP; the permselectivity trend can be attributed to the lower hydration numbers of AN-alkylated membranes, 8.2 (AN) vs. 9.6 (NMP). The conductivity of AN-alkylated membranes is slightly lower due to the lower ion-exchange capacity and hydration numbers.

#### 3.4.3. Various Reinforcing Materials: Membrane Defectiveness

The variation of the reinforcing material has a small impact on conductivity and potentiostatic transport numbers (Table S4). The observed differences in permselectivity are close to the error limit. In the case of conductivity, there is a correlation with the porosity of the reinforcing mesh: higher conductivity was observed for more porous materials, PA mesh, PP mesh, and non-woven PET, which is expected since the conductive polymer fills the free pore space of the reinforcing mesh, increasing the diffusion path [47,48].

However, the most critical feature of the reinforcing material selection is the defectivity of the membranes. For most of the study, about 30 samples were made on PA mesh, 6 of which were defective (20%), and as a result, the samples had to be remade. If the defect was large, a leak was observed when a differential fluid pressure was created, signaling the presence of a through hole. A much more sensitive parameter allowing the tracing of large and small defects was diffusion permeability–which for ordinary membranes is small because of their high permselectivity and is in the range of 2 × 10^−8^–6 × 10^−7^ cm^2^/s, but for defective materials, increases above 1 × 10^−5^ cm^2^/s. The second most frequently used was non-woven PP; among 15 samples, none showed abnormal diffusion permeability or any leakage. The other reinforcing materials were used to synthesize 1–2 membranes, among which there were no defective ones, although such a small selection is insufficient to conclude defect resistance. The proposed mechanism of the influence of reinforcing fabric on defectiveness is discussed in Supplementary Materials (part 2.3, Figure S7).

#### 3.4.4. Selectivity-Conductivity Trade-Off and Comparison with Other Membranes

**Figure 12:**
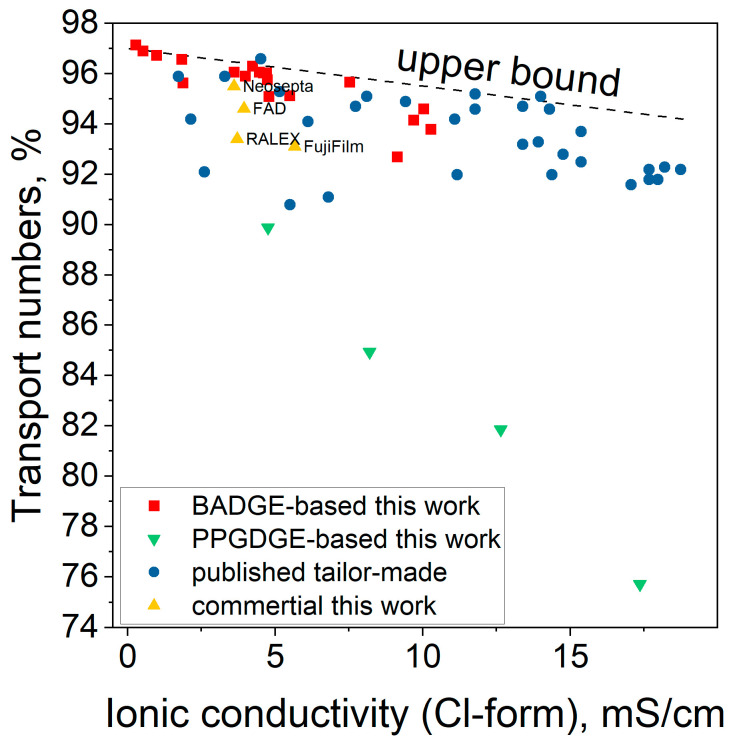
Correlation between potentiostatic transport numbers and specific conductivity of synthesized membranes based on BADGE/PPGDGE, the commercial materials tested in this work, previously published data [32,54,55], normalized to the conductivity and permselectivity of Neosepta© AMX.

The correlation dependence of permselectivity on conductivity was plotted (Figure 12) to compare the obtained membranes with commercial materials and tailor-made membranes from other works. While developing new materials is aimed at seeking higher values of conductivity and permselectivity of membranes simultaneously, there is typically a trade-off between these two parameters: the higher the water content, the higher the conductivity, but the lower the permselectivity. This behavior can be tracked based on different types of ion exchange membrane materials and explained by the fundamental nature of these properties [49,50,51,52,53].

Some patterns were observed for the developed materials, which affected their position on the plotted correlation. Firstly, for both BADGE and PPGDGE-based membranes, the higher the proportion of PEI and inert solvent, the lower the permselectivity and the higher the conductivity of the membranes, which is expected and associated with increasing water uptake. Secondly, PPGDGE-based materials are less efficient than BADGE-based materials—their permselectivity is much lower for the same conductivity values. This is explained by the excessive hydration of PPGDGE-based materials, which is 3–4 times higher than BADGE-based materials. Perhaps non-selective ion transport can occur across the hydrated but uncharged part of the membrane, which consists of PPGDGE.

The efficiency of BADGE-based membranes in the region of high selectivities and low or medium conductivities is higher than that of some of the most superior commercially available materials Neosepta and FAD, or tailor-made membranes obtained in other works. BA_5-30 materials with transport numbers 95–96% and conductivity of 4.8–5.0 mS/cm close to the commercial membrane Neosepta AMX can be considered optimal for electrodialysis desalination.

#### 3.4.5. Stress-Test Stability in Acidic, Alkaline and Neutral Media

**Figure 13:**
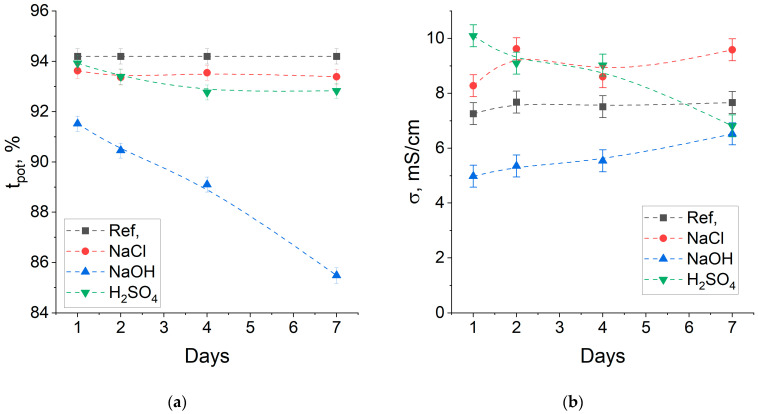
Relationship between the transport numbers (**a**) and conductivity (**b**) on the exposure time.

The stability of anion exchange membranes in acidic and alkaline environments largely determines the processes in which they can be used. To investigate the stability, 3 samples of BA_7-30_PA_ACE membranes were exposed to 5% m/m solutions of sodium chloride (NaCl), sodium hydroxide (NaOH) and sulfuric acid (H_2_SO_4_) at 70 °C. The permselectivity of membranes kept in NaOH dropped significantly during the test (Figure 13). The conductivity of the membranes changed slightly, with a slight increase in values for NaCl and NaOH membranes and a decrease for H_2_SO_4_.

Water uptake and capacity were measured after 7 days of the test (Table 4). The permselectivity of the NaOH membrane decreased due to increasing water uptake and decreasing degree of quaternization (falling s-IEC and increasing w-IEC). Both these phenomena occur due to the degradation of functional groups in an alkaline medium, which is well-known for most anion-exchange membranes [56]. At the same time, some increase in conductivity despite a drop in capacity is associated with an increase in water uptake. For the NaCl-treated membrane, a slight drop in permselectivity and increase in conductivity relative to the reference membrane is related to an increase in water uptake, likely caused by heating. The increase in water uptake during heating is a well-known phenomenon attributed to the rise in the mobility of polymer chains and structure rearrangement [57,58]. Some decrease in permselectivity and conductivity for the H_2_SO_4_-treated membrane is probably due to a reduction in the degree of quaternization. The mechanism of acidic degradation of membranes is less studied than alkaline degradation, but despite the harsh conditions, the membrane showed good acidic stability. Thus, the analyzed membranes are not recommended for use only in strongly basic media due to alkaline degradation.

### 3.5. Mechanical Properties

**Figure 14:**
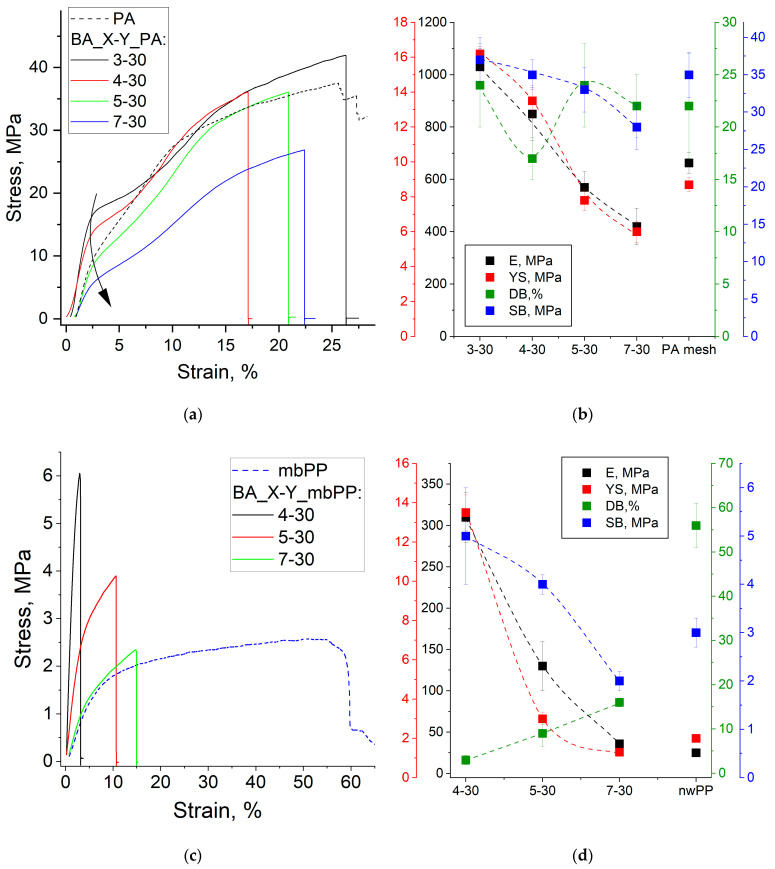

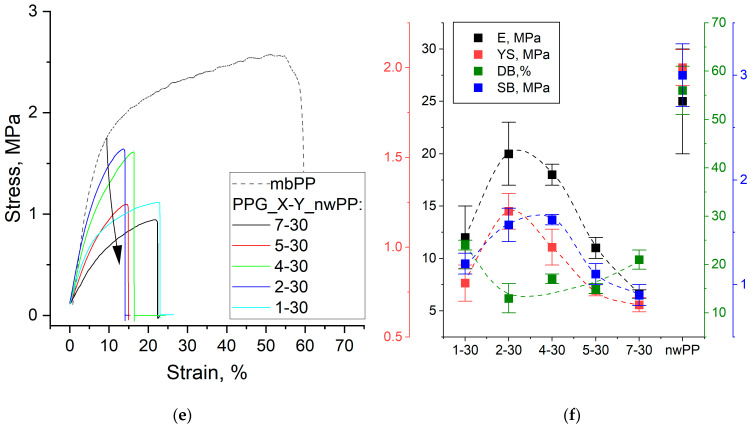
Some stress–strain curves (**a**,**c**,**e**) and average mechanical parameters (**b**,**d**,**f**) for BA_X-Y _PA membranes (**a**,**b**), BA_X-Y_nwPP (**c**,**d**) and PPG_X-Y_nwPP.

The mechanical characteristics of membranes depend on both the copolymer composition and the reinforcing material [47,48]. For developed membranes based on high modulus PA mesh, increasing the PEI content decreases the Young’s modulus (E) from 1030 to 420 MPa and the yield strength (YS) from 16.2 to 6.0 MPa, while the deformation at break (DB) and strength at break (SB) remain high in the range of 17–25% and 28–37 MPa, respectively (Figure 14a,b). Even for materials with high PEI content, all characteristics remain at a level close to the characteristics of the reinforcing mesh. This means that the epoxy copolymer contributes to the mechanical properties for BA_3-30 and BA_4-30 membranes, determining rather high values of E and YS. However, the reinforcing material determines the mechanical properties of BA_5-30 and BA_7-30 membranes. Such an increase in the elasticity with increasing PEI fraction is associated with increasing water content. Water for hydrophilic polymers, including ion-exchange polymers, acts as a low molecular weight plasticizer, increasing elasticity by increasing polymer chains’ mobility [59].

For membranes based on low-modulus reinforcing material-non-woven PP, similar patterns are observed in the series BA_4-30, BA_5-30, BA_7-30, but since the material itself has low E, YS and SB compared to PA mesh, all membrane characteristics are lower (Figure 14c,d). For the other membranes based on different reinforcing matrices, the performance is also proportional to the characteristics of the reinforcing materials, as shown for BA_5-30 membrane (Supplementary, Table S5).

PPGDGE-based membranes have the lowest mechanical performance (Figure 14e,f) due to their extremely high degree of hydration. However, these properties are sufficient to successfully test electrochemical properties without damaging them. Not counting membranes PPG_1-30 with a low degree of polymerization, a drop in E and YS and an increase in DB are also observed with increasing PEI fractions for such membranes.

### 3.6. Diffusion Permeability of Acids and Heavy Metals

**Figure 15:**
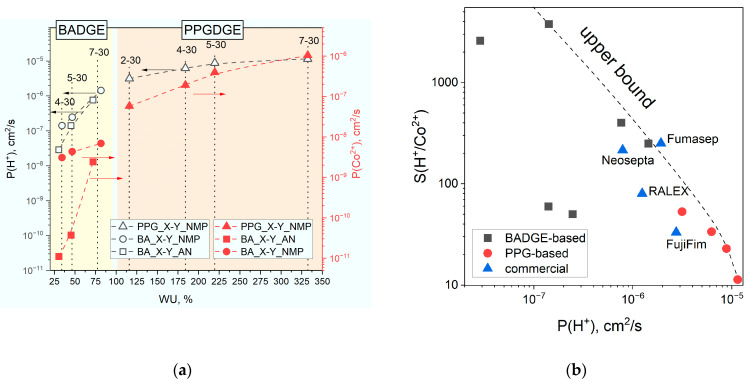
Correlations between proton and metal permeability and water uptake (**a**) and H^+^/Co^2+^-selectivity (**b**).

Since the developed membranes showed good stability in an acidic medium, we investigated the permeability of nitric acid and heavy metal salts Co^2+^ and Zn^2+^ to evaluate their applicability to diffusion dialysis. It turned out that, as well as for selectivity in sodium chloride, the key factor for permeabilities is membrane water uptake, which is seen from the plotted correlation (Figure 15). In this regard, the permeabilities for PPGDGE-based membranes are higher than for BADGE, and the permeabilities for membranes alkylated in NMP are higher than in NMP. 

The correlations for zinc are the same, and data are reported in Supplementary (Figure S8). However, features of membranes alkylated in AN compared to NMP are observed—lower acid and metal permeability values for membranes 4-30 and 5-30. This is especially evident for the heavy metal permeability, which is 2–3 orders of magnitude lower, providing record values of H^+^/Co^2+^-selectivity coefficients above 2000. This feature is probably due to the large number of weakly basic amino groups (relatively low degree of quaternization) of the membranes alkylated in AN, which may increase the selectivity due to the slowing down of transport due to complexation between amino groups and cobalt, zinc or proton atoms. This is also indirectly indicated by the fact that there is no such feature for sodium chloride permeability (Figure S8) since complexes with amines are not characteristic of sodium cations, and the permeability of membranes with a lower degree of quaternization is even slightly higher. In addition, it is known that the proton permeability of membranes with weakly basic groups is much lower, which is used in materials with proton-blocking properties specialized for the recovery of acids by the ED process, e.g., Neosepta© AID [60].

The trade-off correlation between selectivity and permeability coefficients can also be plotted for diffusion dialysis to estimate the material efficiency. BADGE-based materials are close in efficiency to the Fumasep-FAD membrane used in industrial diffusion dialysis modules [61]. BA_5-30_AN membrane has a relatively small permeability of 1.4 × 10^−7^ cm^2^/s but demonstrated a record H^+^/Me^2+^-selectivity value of 3800. Membranes based on PPGDGE, on the contrary, are highly permeable (1 × 10^−5^ cm^2^/s) and low selective with S(H^+^/Me^2+^) of 10–30. BA_7-30_NMP membrane with a permeability of 1.44 × 10^−6^ cm^2^/s (U(H^+^) ≈ 36 m/h) and S(H^+^/Me^2+^) of 208 (Co) and 250 (Zn) close to the commercial Fumasep FAD membrane can be considered as optimal for DD process. It is also worth mentioning membrane BA_4-30_AN has almost two orders of magnitude lower acid permeability coefficient of 3 × 10^−8^ cm^2^/s, and due to proton blocking properties, this material may be used for recovery of acids by ED process.

### 3.7. Current-Voltage Characteristic

**Figure 16:**
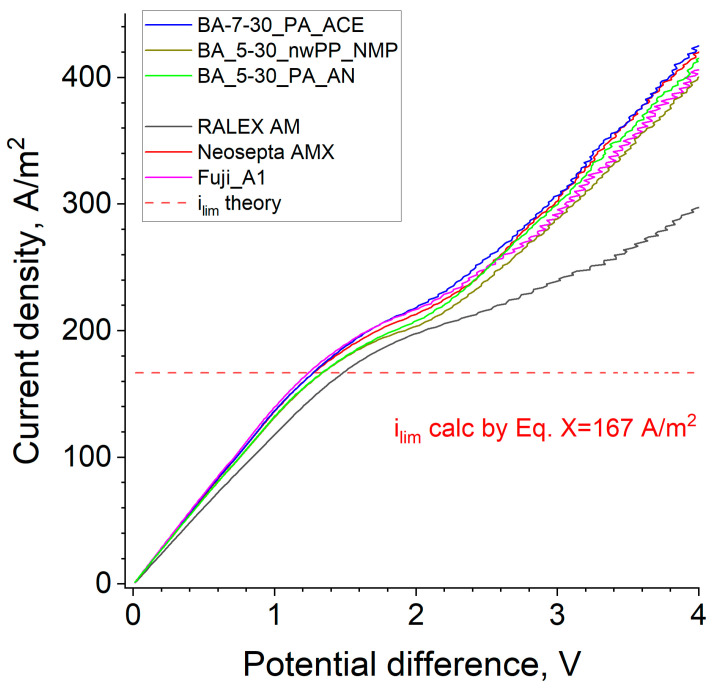
Current-voltage characteristics of some developed and commercial membranes: 0.1 M NaCl, linear flow rate is 0.72 cm/s.

The efficiency of the ion exchange membrane in the ED-desalination process can be evaluated by the characterization of basic membrane properties such as conductivity and permselectivity and by studying its current-voltage characteristic (CVC) in the cell simulating an electrodialysis module. In the first region of CVC (Figure 16), the current grows almost linearly with voltage, which is consistent with Ohm’s law—the total ionic resistance of the solutions and the membrane determines the slope of the curve in this region [62].

Table 5 summarizes the calculated ionic resistance values from the linear CVC sections and the membrane resistances obtained by subtracting the resistance of the cell without membrane (66.8 Ω cm^2^). Although the membrane resistance values determined by this method are much less accurate than those determined by direct measurement because the membrane contribution to the system resistance is only 4–10%, the data obtained correlate well with the conductivity measured by the contact method. The synthesized membranes have low ionic resistance compared to commercial membranes Neosepta^®^ AMX and FujiFilm^®^ A1 and are much lower than the heterogeneous RALEX^®^ membrane (Table 5).

With further increases in voltage, when reaching a limiting current, the linear dependence of current on voltage starts to decline towards lower currents. In this second region, the concentration of the solution near the membrane surface approaches zero, the resistance of the system grows, and the current is mainly determined by the diffusion rate of the electrolyte to the membrane surface [33,63]. The theoretical limiting current value (Equation (9)) is 167 A/m^2^, while the experimental values are 10–20% higher. According to [34], the observed difference is due to the contribution of gravitational convection arising from the difference in density of 0.1 M sodium chloride solution and desalinated water near the membrane surface. The i_lim_ of the developed membranes is very close to the values of commercial materials. After the limiting current plateau, the current starts to increase again, which is associated with the development of electroconvection [64]. In this area, the obtained membranes are close to commercial samples of homogeneous membranes.

## 4. Conclusions

Novel self-standing anion-conducting membranes based on epoxy resins were obtained. The synthesis involves the copolymerization of epoxy resin components, namely polyethyleneimine and bisphenol A diglycidyl ether or poly(propylene glycol) diglycidyl ether, followed by PEI quaternization to introduce charged groups. The advantages of the proposed approach include the absence of complex chemical reactions, consequently minimal use of organic solvents and high availability of starting reagents/materials. By varying the composition of the ternary reaction mixture (diepoxide, polyethyleneimine, diluent) and the chemical structure of the diepoxide, it is possible to control the ion exchange capacity of the membranes in the range of 1.5–3.7 mmol/g and the water uptake of the membranes in the range of 20–92% for bisphenol A diglycidyl ether and 110–330% for poly(propylene glycol) diglycidyl ether. In particular, increasing the PEI/diepoxide ratio increases capacity and water uptake, while increasing the diluate content leads to an increase in water uptake. The PEI quaternization degree depends on the choice of solvent for the quaternization reaction; for good solvents such as NMP or ACE, the quaternization degree is higher than for bad solvents such as AN.

The choice of reinforcing material has been found to impact the mechanical properties and defectiveness but not other membrane properties. The higher the toughness and strength of the reinforcing mesh, the higher the corresponding properties of the membranes. However, the defect-free membranes with low salt permeability values were obtained using low-modulus, non-woven polypropylene fabric.

The chemical stress-test study has illustrated that the synthesized membranes exhibit excellent stability in neutral and acidic media. However, they gradually lose ion exchange capacity and permselectivity in sodium hydroxide media.

Varying the ion exchange capacity and water content allows obtaining materials with conductivity in the range of 0.2 and 17 mS/cm (Cl form, 20–22 °C) and potentiostatic transport numbers in the 75 to 97% range. For all materials, conductivity increases, but permselectivity decreases with increasing membrane water uptake and ion exchange capacity. Because of the optimal degree of hydration, membranes based on hydrophobic BADGE have a much better conductivity/selectivity trade-off than membranes based on hydrophilic PPGDGE. Comparison with commercial materials such as Neosepta, RALEX, Fujifilm, and tailor-made membranes demonstrated that BADGE-based materials are comparable to or better than the best-known anion exchange membranes in permselectivity and conductivity.

Finally, the optimized membranes were tested for recovering acid by diffusion dialysis and for NaCl desalination by CVC measurements. BA_7-30_NMP membrane has a permeability for nitric acid of 1.4 × 10^−6^ cm^2^/s (U(H+) ≈ 36 m/h) and selectivity H^+^/Co^2+^ and H^+^/Zn^2+^ 200–250, which is similar to commercial Fumasep FAD-75 membrane. According to the current-voltage characteristics of a laboratory electrodialysis cell, the designed membranes have a relatively low area resistance (3–6 Ω cm^2^) and a high limiting current (170–190 A/m^2^) close to commercial materials, which, together with high stability and good mechanical properties, indicates the applicability of the developed membranes in the electrodialysis desalination.

## Figures and Tables

**Table 1:** Maximum heat flux, curing end time, and total heat released divided by the number of mole epoxide groups for BADGE or PPGDGE-based copolymers of different compositions.

Composition	Max Heat Flow, mW/mg	Reaction Time, min	Total Heat Per Epoxide Group, kJ/mole
BA_3-20	−2.00	46	113
BA_3-30	−1.45	59	99
BA_3-40	−0.925	83	96
BA_5-20	−2.77	26	112
BA_5-30	−2.02	26	109
BA_5-40	−1.72	37	116
BA_7-20	−3.08	21	113
BA_7-30	−2.75	30	102
BA_7-40	−1.79	44	101
PPG_1-30	−0.43	138	51
PPG_2-30	−0.78	92	87
PPG_4-30	−1.03	70	116
PPG_5-30	−1.06	62	116
PPG_7-30	−1.26	50	121

**Table 2:** Degree of swelling (%) for BA_7-30 membrane before and after quaternization.

Solvent	Non-Quaternized	Quaternized
water	39.3	74.2
ACE	8.3	3.3
AN	4.8	1.7
NMP	72.7	6.7
TCM	65.4	4.1

**Table 3:** Ion exchange capacity, a fraction of weak-base amino groups, water uptake, conductivity, and transport numbers values for membranes alkylated in different solvents.

Membrane	Solvent	s-IEC ± 0.05, mmole/g	w-IEC ± 0.05, mmole/g	Weak-Base Amino Group Fraction, %	WU, %	σ (Cl Form, 20–22 °C) ± 0.5, mS/cm	t_pot_ ± 0.3, %
BA_7-40_PA	ACE	3.45	1.31	27	92.2	10.3	92.7
BA_7-40_PA	AN	2.80	1.45	34	87.9	8.9	92.2
BA_7-40_PA	TCM	2.90	1.33	32	81.5	10.0	92.2
BA_7-40_PA	NMP	Sample collapse due to high swelling					
BA_7-30_nwPP	AN	2.55	2.34	48	71.8	9.1	94.2
BA_7-30_nwPP	NMP	3.10	1.59	34	81.7	9.7	92.7

**Table 4:** Water uptake and ion-exchange capacity values for BA_7-30_PA_ACE membranes after a 7-day stress test at 70 °C.

Solution	WU, %	s-IEC, mmole/g	w-IEC, mmole/g
Reference	70	2.70	1.50
5% m/m NaCl	83	2.70	1.45
5% m/m NaOH	130	1.40	2.80
5% m/m H_2_SO_4_	74	2.30	1.85

**Table 5:** DC resistance of the cell and membranes and limiting currents obtained from CVC treatment.

Membrane	R_cell_ ± 0.8, Ω cm^2^	R_mem_ ± 1.2, Ω cm^2^	i_lim_, A/m^2^
BA_5-30_PA_AN	73.1	6.3	177
BA_5-30_nwPP_NMP	73.4	6.6	171
BA_7-30_PA_ACE	70.1	3.2	179
RALEX^®^ AM	81.8	15.0	174
Neosepta^®^ AMX	71.2	4.4	179
FujiFilm^®^ T1	69.4	2.6	185

## Data Availability

The original contributions presented in the study are included in the article and supplementary materials, further inquiries can be directed to the corresponding author.

## Supplementary Materials

The following supporting information can be downloaded at: https://www.mdpi.com/article/10.3390/membranes14060138/s1, Figure S1: Typical time dependences of the acid concentration in the diluate chamber calculated from the conductivity of the solution. Figure S2: DSC curves for PPGDGE-PEI of different compositions. 80 °C isotherm. Figure S3: (a) Isothermal curing DSC curves at different temperatures for BA_7-30 copolymers, (b) dependence of the maximum rate in Arrhenius coordinates for BA_7-30 copolymers. Figure S4: Series of images of initial reinforcing meshes and membranes of BA_5-30 composition based on them: non-woven PET (an), mesh PET (bn), non-woven PP (cn), mesh PP (dn). *n* = 1—SEM images of initial meshes; *n* = 2, 3—SEM images of membranes; *n* = 4—optical image of membranes. Figure S5: SEM images of the cross-section and surface of the BA_5-30_PA_ACE membrane. Figure S6: SEM images of PPGDGE-PEI based membranes: 1-30 (a), 2-30 (b), 4-30 (c), 5-30 (d), 7-30 (e). Figure S7: Defect formation scheme for the high defectivity of PA mesh-based and low nwPP-based mem-branes and SEM images of the starting materials for PA mesh and non-woven PP: 1—water, 2—crosslinked PEI phase, 3—reinforcing polymer. Figure S8: Dependence of diffusion permeability of zinc nitrate (a) and sodium chloride (b) on water uptake of membranes. Table S1: Labels and typical masses of ternary mixture components. Table S2: Maximum heat flux, curing end time, and total heat released divided by the number of mole epoxide groups for BA-7-30 copolymer for different curing temperatures. Table S3: Stationary and transport properties of BA_7-30_PA_ACE membranes polymerized at different temperatures. Table S4: Conductivity, transport numbers and sodium chloride permeability of BA-5-30 membranes based on different reinforcing materials and porosity of these materials. Table S5: Stress-strain characteristic properties of reinforcement materials. References [35,36] are cited in Supplementary Materials.

## Abbreviations

AEM	Anion exchange membrane
AN	Acrylonitrile
ACE	Acetone
BADGE or BA	Bisphenol A diglycidyl ether
DB	Deformation at break
DD	Diffusion dialysis
E	Young’s modulus
ED	Electrodialysis
IEC	Ion-exchange capacity
mPET	Polyethylene terephthalate
mPP	Polypropylene mesh
NMP	N-methylpyrolidone
nwPET	Non-woven polyethylene terephthalate fabric
nwPP	Non-woven polypropylene fabric
PA	Polyamide
PEI	Polyethyleneimine
PPGDGE or PPG	Poly(propylene glycol) diglycidyl ether
SB	Strength at break
TCM	Trichloromethane
WU	Water uptake
YS	Yield strength
