# Supplementary material for: Novel Crosslinked Anion Exchange Membranes Based on Thermally Cured Epoxy Resin: Synthesis, Structure and Mechanical and Ion Transport Properties
Source: Membranes (Basel). 2024 Jun 11;14(6):138. doi: 10.3390/membranes14060138 (PMC11205850; doi:10.3390/membranes14060138)
Supplement: Supplementary file 1 [file membranes-14-00138-s001.zip › membranes-3005792-supplementary.pdf]

membranes

Supplementary Materials

Novel Crosslinked Anion Exchange Membranes Based on Thermally Cured Epoxy Resin: Synthesis, Structure and Mechanical and Ion Transport Properties

Daniil Golubenko, Farah Ejaz Ahmed and Nidal Hilal *

New York University Abu Dhabi Water Research Center, New York University Abu Dhabi, P.O. Box 129188 Abu Dhabi, United Arab Emirates
* Correspondence: nidal.hilal@nyu.edu

1. Experimental

Table S1. Labels and typical masses of ternary mixture components.

Label	m (diepoxide), g	m (PEI), g	m (NMP), g
1-30	1.00	0.10	0.55
2-30	1.00	0.20	0.58
3-20	1.00	0.30	0.39
3-30	1.00	0.30	0.65
3-40	1.00	0.30	1.04
4-30	1.00	0.40	0.70
5-20	1.00	0.50	0.45
5-30	1.00	0.50	0.75
5-40	1.00	0.50	1.20
7-20	1.00	0.70	0.51
7-30	1.00	0.70	0.85
7-40	1.00	0.70	1.36

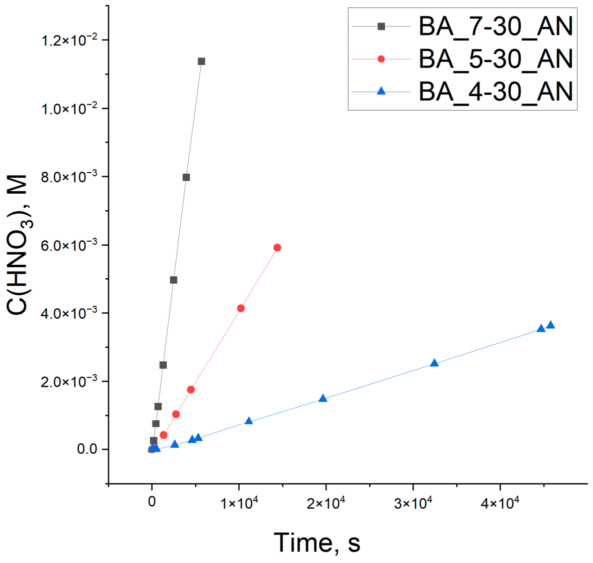

Figure S1. Typical time dependences of the acid concentration in the diluate chamber calculated from the conductivity of the solution.

2. Results and Discussion

2.1. DSC study of curing reaction

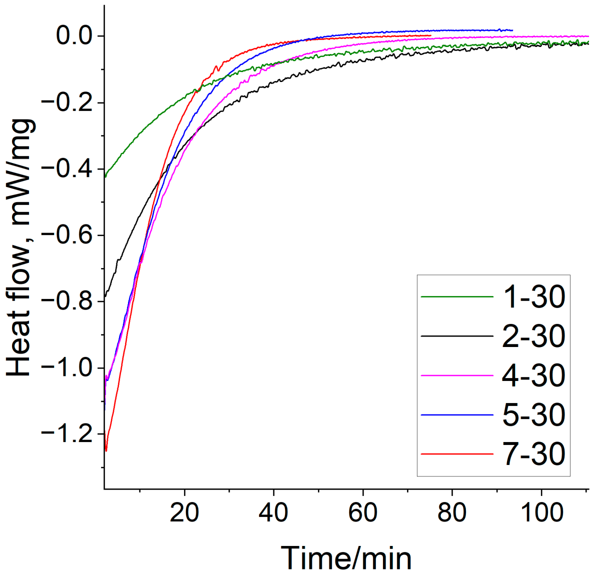

Figure S2. DSC curves for PPGDGE-PEI of different compositions. 80 °C isotherm.

2.2. Temperature curing optimization

Reducing the curing temperature from 80 °C to 50 °C decreases the maximum heat flow from −2.75 to −0.284 mW/mg, and increases the reaction time from 30 to 178 min (Table S2). The value of activation energy obtained from the temperature dependence of the maximum rate in Arrhenius coordinates is 67 ± 10 kJ/mol for the composition 7-30 (Figure S3) and agrees well with literature data for BADGE-based epoxy resins [1,2].

Table S2. Maximum heat flux, curing end time, and total heat released divided by the number of mole epoxide groups for BA-7-30 copolymer for different curing temperatures.

Temperature	Max Heat Flow, mW/mg	Reaction Time, min	Total Heat per Epoxide Group, kJ/mole
50°C	-0.284	178	126
60°C	-0.585	82	118
70°C	-0.869	49	110

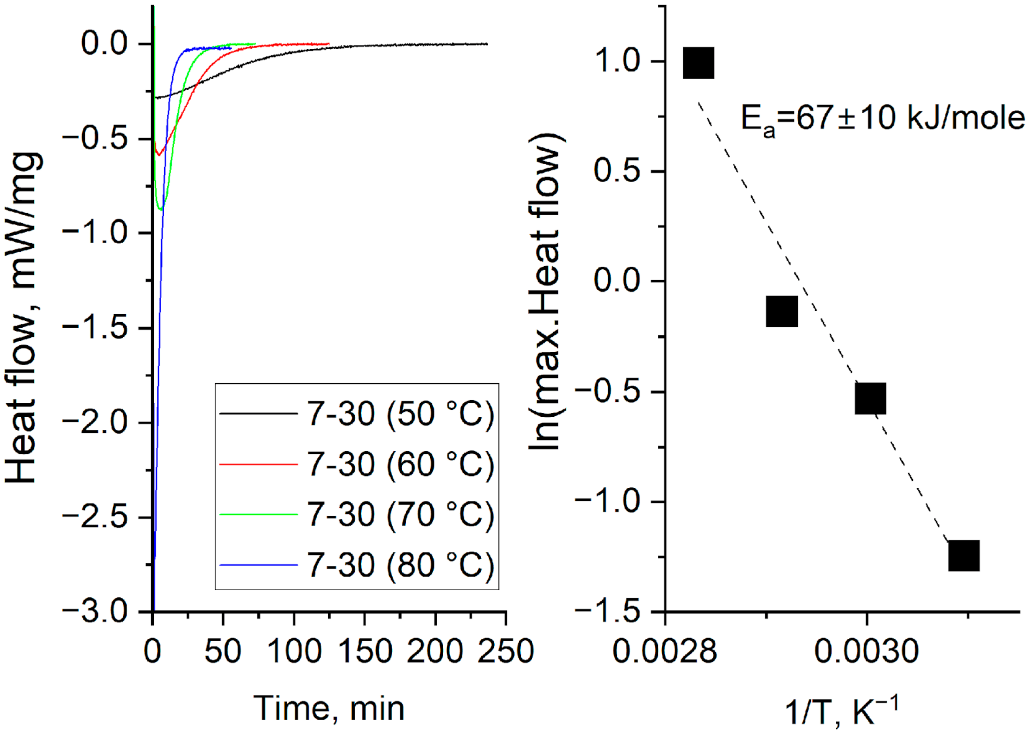

(a) (b)

Figure S3. (**a**) Isothermal curing DSC curves at different temperatures for BA_7-30 copolymers, (**b**) dependence of the maximum rate in Arrhenius coordinates for BA_7-30 copolymers.

Varying the curing temperature in the 50–80 °C range has no systematic effect on the ion exchange capacity of the membranes, water uptake and other transport characteristics, and the observed deviations can be explained by synthesis reproducibility in different runs (Table S3).

Table S3. Stationary and transport properties of BA_7-30_PA_ACE membranes polymerized at different temperatures.

Curing Temperatures	s-IEC ± 0.05, mmole/g	Water uptake ± 3, %	σ, mS/cm	t_{pot} ± 0.3, %
80 °C	3.30	79	10.0±0.5	94.6
70 °C	3.00	72	8.1±0.4	94.7
60 °C	2.95	77	9.0±0.5	94.6
50 °C	2.95	72	7.8±0.4	94.5

2.3. Membrane morphology and composition

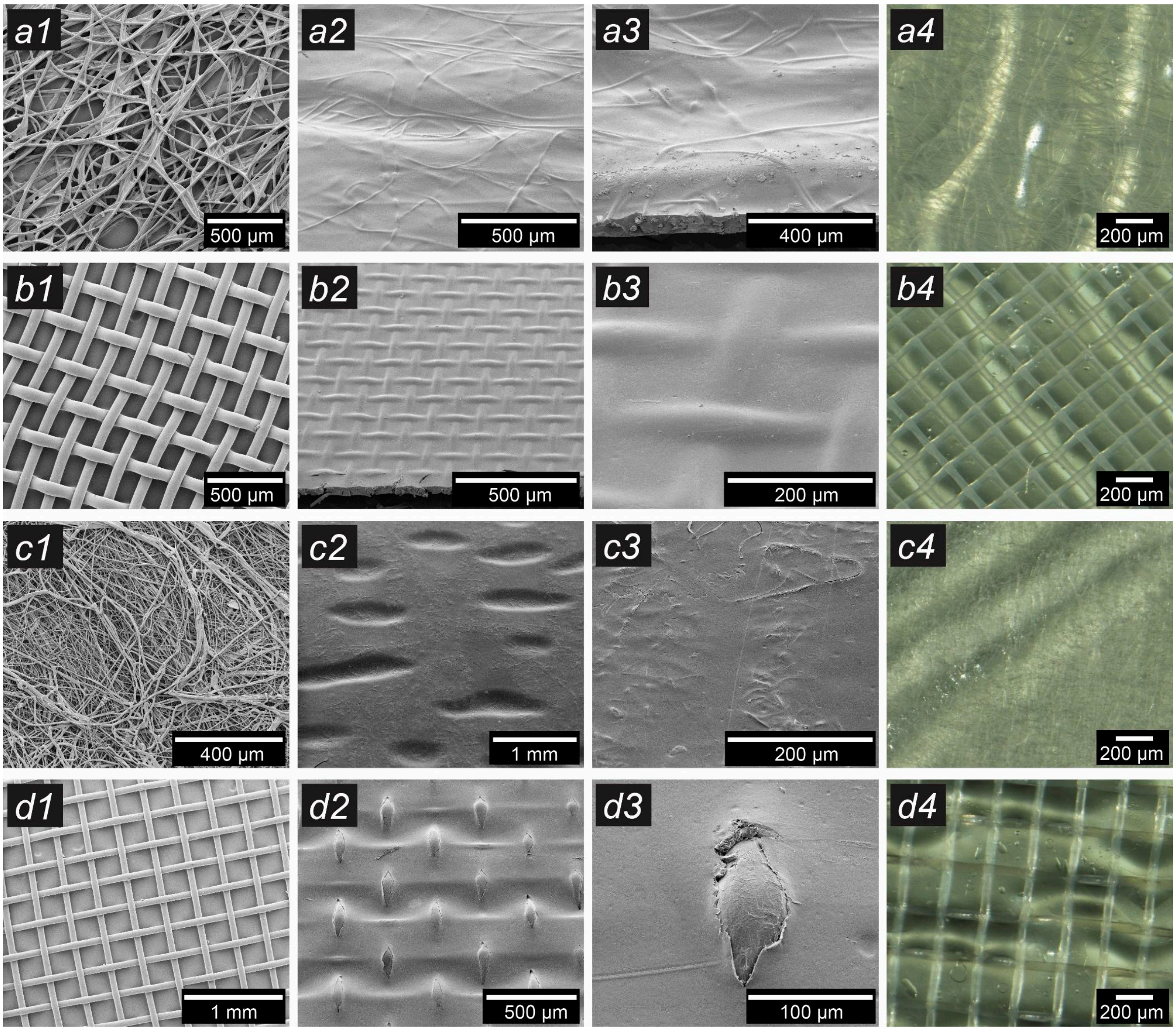

Figure S4. Series of images of initial reinforcing meshes and membranes of BA_5-30 composition based on them: non-woven PET (a*n*), mesh PET (b*n*), non-woven PP (c*n*), mesh PP (d*n*). *n* = 1 - SEM images of initial meshes; *n* = 2, 3 - SEM images of membranes; *n* = 4 - optical image of membranes.

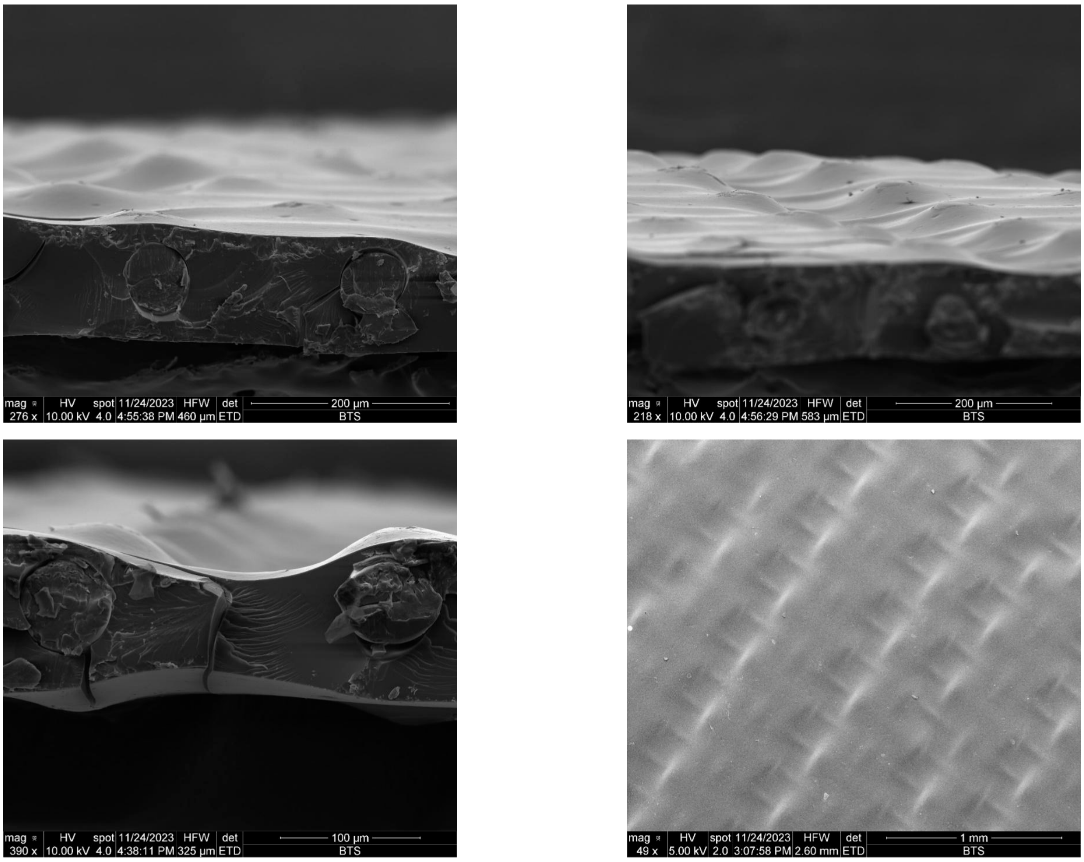

Figure S5. SEM images of the cross-section and surface of the BA_5-30_PA_ACE membrane.

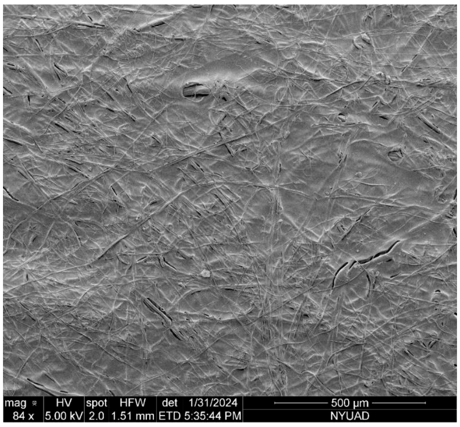

a1

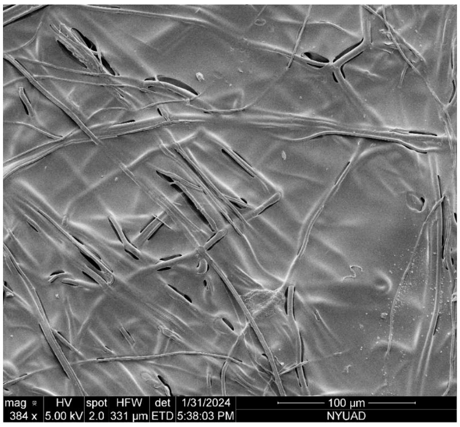

a2

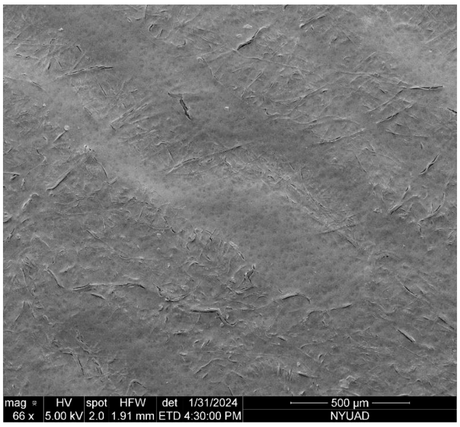

b1

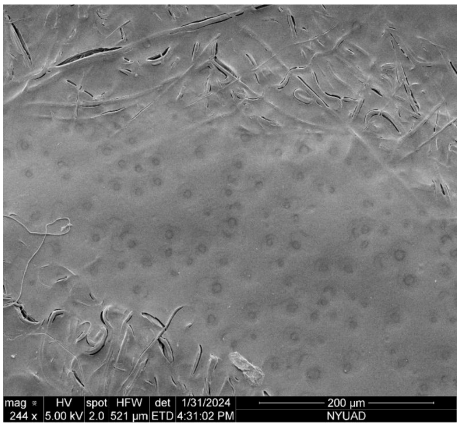

b2

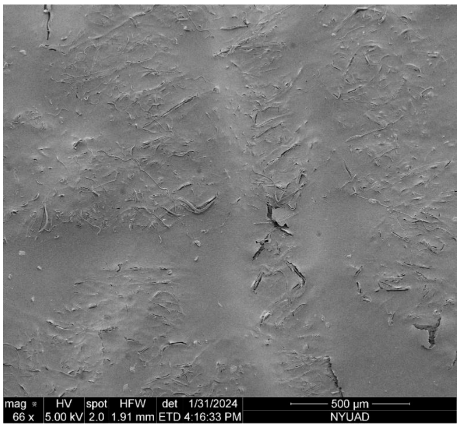

c1

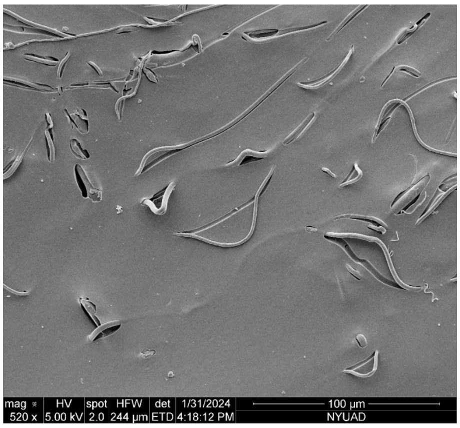

c2

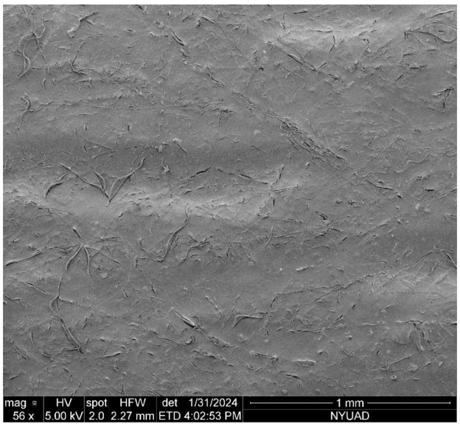

d1

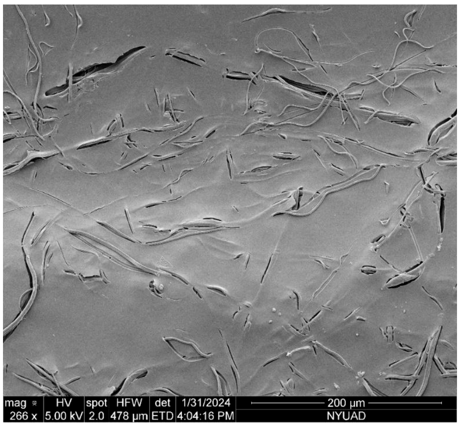

d2

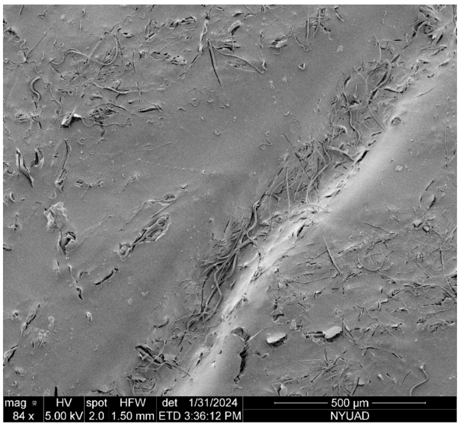

e1

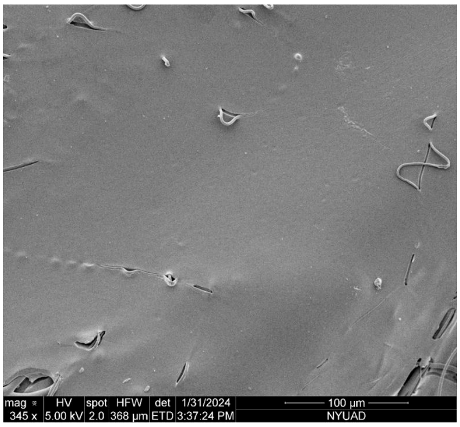

e2

Figure S6. SEM images of PPGDGE-PEI based membranes: 1-30 (**a**), 2-30 (**b**), 4-30 (**c**), 5-30 (**d**), 7-30 (**e**).

2.4. Various reinforcing materials

Table S4. Conductivity, transport numbers and sodium chloride permeability of BA-5-30 membranes based on different reinforcing materials and porosity of these materials.

Reinforcing Material	σ ± 0.2, mS/cm	t_{pot} ± 0.3, %	$P_{NaCl} \times 10^8$, cm²/s	Porosity, %
PA mesh	4.7	95.8	5.5	78
Non-woven PP	4.0	95.9	4.0	73
PP mesh	4.6	96.0	5.0	81
PET mesh	4.2	96.3	3.6	74
Non-woven PET	4.7	96.0	5.9	84

We assume that the critical location of defect formation for PA mesh is the copolymer-filament boundary and the crossing of the reinforcing material filaments (Figure S7). At the crossing point, the copolymer layer is a few microns thick (Figure 7d). If the filament peels away from the copolymer and the micron layer of copolymer above the filament on both sides is defective, a continuous macro-defect is formed through the entire membrane thickness. We have two hypotheses as to why nwPP was resistant to the described defect formation: the first is the low probability of the formation of a continuous defect due to a large number of filament crossings, and the second is that even if the copolymer peels from the filament, a micro-slit is formed, one of the walls of which is a hydrophobic polymer – i.e. such a micro-slit is not filled with liquid and does not create a non-selective

transport channel. Unfortunately, the defectivity of ion exchange membranes is rarely discussed in the literature, so comparison with published results is difficult.

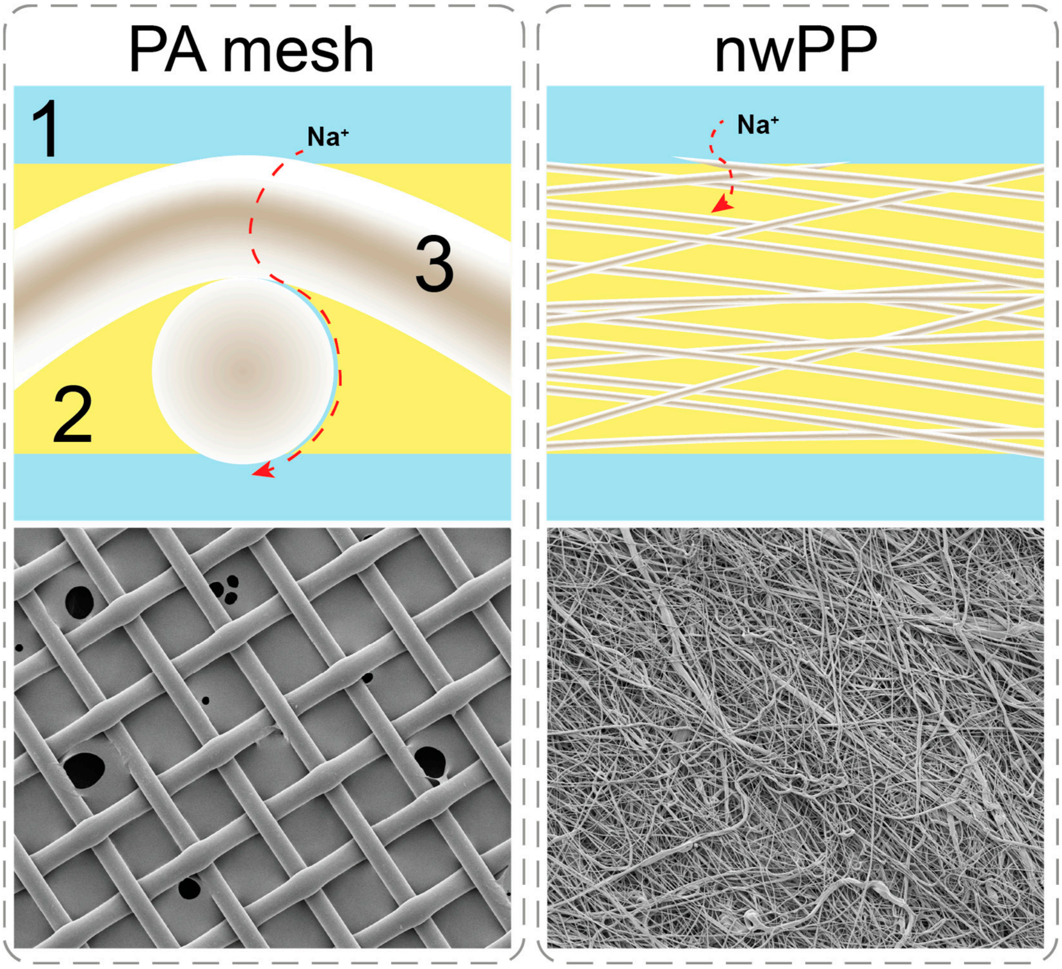

Figure S7. Defect formation scheme for the high defectivity of PA mesh-based and low nwPP-based membranes and SEM images of the starting materials for PA mesh and non-woven PP: 1 - water, 2 - crosslinked PEI phase, 3 – reinforcing polymer.

2.5. Mechanical properties

Table S5. Stress-strain characteristic properties of reinforcement materials.

	**mPA**	**mPP**	**nwPP**	**mPET**	**nwPET**
Deformation at break, %	22 ± 5	39 ± 2	56 ± 5	26 ± 4	29 ± 4
Tensile strength, MPa	35 ± 3	43 ± 2	3.0 ± 0.3	76 ± 6	1 ± 0.2
Young's modulus	660 ± 40	200 ± 20	25 ± 5	720 ± 30	300 ± 20
Yield strength, MPa	8.7 ± 0.4	2.5 ± 0.2	2.0 ± 0.1	12.4 ± 1.2	4.1 ± 0.2

2.6. Acid/Heavy Metals Transport

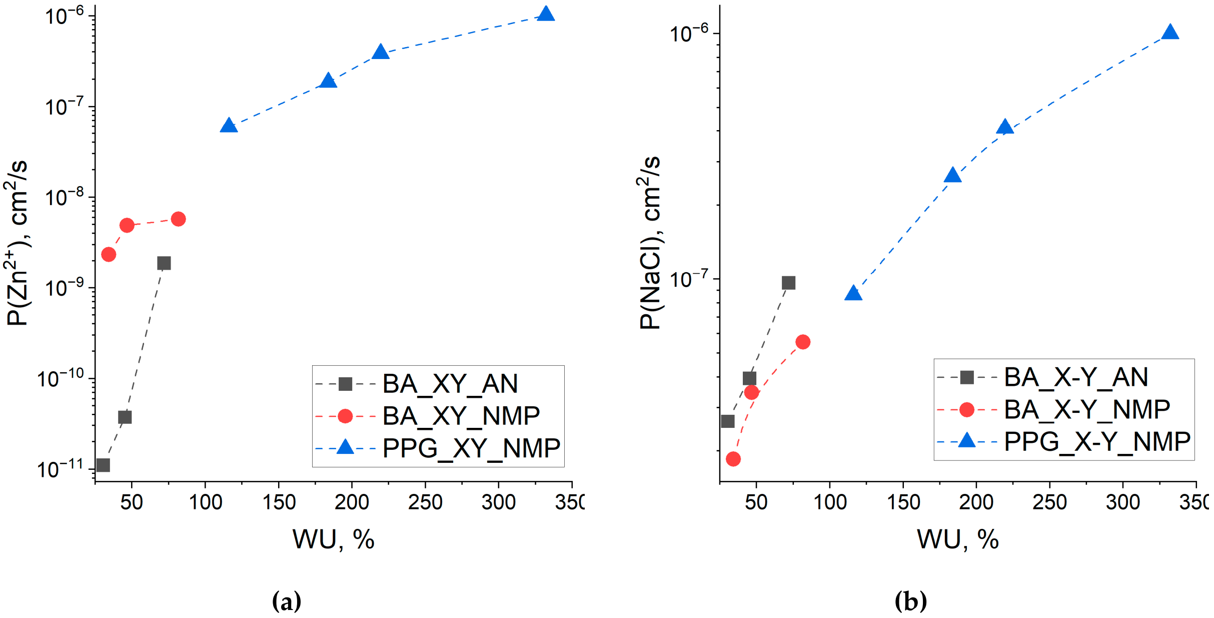

Figure S8. Dependence of diffusion permeability of zinc nitrate (**a**) and sodium chloride (**b**) on water uptake of membranes.

References:

1. Severini, F.; Gallo, R.; Marullo, R. Differential Scanning Calorimetry Study of Reactions of Epoxides with Polyamines. *J. Therm. Anal.* **1982**, *25*, 515–523. https://doi.org/10.1007/BF01912977.
2. Santiago, D.; Fernández-Francos, X.; Ramis, X.; Salla, J.M.; Sangermano, M. Comparative Curing Kinetics and Thermal–Mechanical Properties of DGEBA Thermosets Cured with a Hyperbranched Poly(Ethyleneimine) and an Aliphatic Triamine. *Thermochim. Acta* **2011**, *526*, 9–21. https://doi.org/10.1016/j.tca.2011.08.016.
